# A Sense of Balance: Experimental Investigation and Modeling of a Malonyl-CoA Sensor in *Escherichia coli*

**DOI:** 10.3389/fbioe.2015.00046

**Published:** 2015-04-08

**Authors:** Tamás Fehér, Vincent Libis, Pablo Carbonell, Jean-Loup Faulon

**Affiliations:** ^1^Institute of Systems and Synthetic Biology, University of Evry Val d’Essonne, Evry, France; ^2^Institute of Biochemistry, Biological Research Centre of the Hungarian Academy of Sciences, Szeged, Hungary; ^3^Paris Diderot University, Paris, France; ^4^Research Programme on Biomedical Informatics (GRIB), Department of Experimental and Health Sciences, Hospital del Mar Medical Research Institute (IMIM), Universitat Pompeu Fabra, Barcelona, Spain; ^5^SYNBIOCHEM Center, Manchester Institute of Biotechnology, School of Chemistry, University of Manchester, Manchester, UK

**Keywords:** malonyl-CoA, dynamic pathway regulation, high-throughput screening, synthetic regulatory circuit, fluorescent reporter circuit, sensor–actuator circuit

## Abstract

Production of value-added chemicals in microorganisms is regarded as a viable alternative to chemical synthesis. In the past decade, several engineered pathways producing such chemicals, including plant secondary metabolites in microorganisms have been reported; upscaling their production yields, however, was often challenging. Here, we analyze a modular device designed for sensing malonyl-CoA, a common precursor for both fatty acid and flavonoid biosynthesis. The sensor can be used either for high-throughput pathway screening in synthetic biology applications or for introducing a feedback circuit to regulate production of the desired chemical. Here, we used the sensor to compare the performance of several predicted malonyl-CoA-producing pathways, and validated the utility of malonyl-CoA reductase and malonate-CoA transferase for malonyl-CoA biosynthesis. We generated a second-order dynamic linear model describing the relation of the fluorescence generated by the sensor to the biomass of the host cell representing a filter/amplifier with a gain that correlates with the level of induction. We found the time constants describing filter dynamics to be independent of the level of induction but distinctively clustered for each of the production pathways, indicating the robustness of the sensor. Moreover, by monitoring the effect of the copy-number of the production plasmid on the dose–response curve of the sensor, we managed to coarse-tune the level of pathway expression to maximize malonyl-CoA synthesis. In addition, we provide an example of the sensor’s use in analyzing the effect of inducer or substrate concentrations on production levels. The rational development of models describing sensors, supplemented with the power of high-throughput optimization provide a promising potential for engineering feedback loops regulating enzyme levels to maximize productivity yields of synthetic metabolic pathways.

## Introduction

Natural metabolic pathways are highly regulated and can adapt dynamically to changes in the levels of chemicals within and around the cell (Chubukov et al., 2014). For this purpose, cells have developed a high number of sensors to monitor and control their own metabolic state. This stringent regulatory process is impaired or lost when metabolic engineers insert heterologous enzymes into artificial pathways, usually at non-optimal levels of activities, leading to accumulation of intermediate metabolites and reduced growth rate (Holtz and Keasling, 2010). To address this issue, several projects have attempted to mimic natural regulation by adding such sensors to engineered metabolic pathways. The advantages of using natural sensors such as transcription factors to implement synthetic dynamic regulation in heterologous pathways have been demonstrated in several studies: following pioneering work on lycopene synthesis regulation (Farmer and Liao, 2000), this strategy also led recently to higher yields in fatty acid synthesis with the help of fatty acids (Zhang et al., 2012) and malonyl-CoA (Xu et al., 2014; Liu et al., 2015) biosensors.

Such biosensor-based approaches allow the bacterial factory to artificially monitor its own level of metabolites and to modify expression levels of certain enzymes in order to balance pathway function. They can also be used to devise external monitoring systems to aid the selection of cells displaying improved biological functions. A major application for this monitoring is the screening of libraries of variant strains in directed evolution of enzymes (Michener and Smolke, 2014). Screening of producers is still a major bottleneck in metabolic engineering as quantification of the compound of interest usually relies on low-throughput measurement techniques such as liquid or gas chromatography. However, the typical throughput necessary for directed evolution applications or combinatorial libraries’ screening is several orders of magnitude higher (Dietrich et al., 2010). Therefore, *in vivo* biosensing offers a relevant non-destructive, cost-effective, and high-throughput alternative for monitoring production levels (Schallmey et al., 2014). This strategy has proven successful in identifying high-producing strains, via the coupling of biosensor to reporter genes such as fluorescent proteins or fitness-related proteins. The screening can either be done at the colony level, in microtiter plates or by fluorescence-activated cell sorting (FACS). For instance, screening of *Escherichia coli* colonies expressing a transcription factor evolved to detect mevalonate allowed successful identification of high producers of mevalonate in a library of mevalonate-synthesis variants (Tang and Cirino, 2011). Theoretically, FACS may allow even higher throughputs and was notably used to evolve the caffeine demethylase enzyme in yeast cells harboring riboswitches able to detect theophylline, the enzyme’s product (Smolke and Michener, 2012). FACS was also used in combination with transcription factor-based sensors to measure l-lysine production in *C. glutamicum* (Binder et al., 2012) and detect production of Kaempferol in engineered *E. coli* (Siedler et al., 2014). Alternatively, growth rate can be coupled to the production of the product of interest via transcription factor-dependent expression of antibiotic resistance proteins. This strategy has been used in a proof of principle experiment to enrich a population of *E. coli* for cells harboring the pathway for 1-butanol-production (Dietrich et al., 2013). Later, the power of multiplex automated genome engineering was combined with this approach to optimize the production of naringenin and glucaric acid in *E. coli* (Raman et al., 2014).

Because both pathway dynamic regulation and screening rely on the same type of biosensors, a synergy exists between these two approaches. Illustrating such synergy, here we adapted a malonyl-CoA biosensor previously developed for a dynamic regulation purpose (Liu et al., 2015) to a new screening goal. Transcription factor-based biosensing is particularly interesting for monitoring malonyl-CoA as it is difficult and time consuming to quantify this molecule *in vivo* via standard LC/MS. In addition to being an important step for fatty acid synthesis in *E. coli*, malonyl-CoA production is also a limiting step for the production of plant flavonoids in engineered *E. coli*. Our group is working on the development of computer-assisted design of metabolic pathways and is seeking high-throughput techniques to test predicted pathways and enzymes for continual feedback-optimization of the prediction algorithm. In our earlier proof of concept work, we used our software RetroPath (Carbonell et al., 2011) to identify and rank pathways capable of producing the high-value flavonoid pinocembrin in *E. coli*. During the implementation of multiple constructs, Malonyl-CoA, was identified as a bottleneck, preventing high-yield production. Here, we used the biosensor based on transcription factor FapR to analyze the malonyl-CoA-producing pathways that were proposed by our software to remove the bottleneck. We identified optimal conditions for malonyl-CoA monitoring by fine-tuning levels of expression of the transcription factor and we fitted our experimental data to a model giving insights on proper interpretation of output profiles encountered in such screening protocols. This approach helped us identify the best candidate pathways, validate two novel routes of malonyl-CoA synthesis, and optimize enzyme-expression levels through modification of plasmid copy-number.

## Materials and Methods

### Strains and plasmids

Plasmid construction was done in *E. coli* strain DH5α, and all malonyl-CoA sensing experiments were carried out in strain BL21DE3. Plasmid pBFR1k_RFP_8FapR (Liu et al., 2015) was a kind gift of Dr. Fuzhong Zhang (Washington University, St. Louis, MO, USA). To construct plasmid pCFR, pBFR1k_RFP_8FapR was PCR-amplified using primers pBFR-FW (ACT*GTCGAC*GAAACGATCCTCATCCTG) and pBFR-Rev (GC*TCTAGA*TTCTTCTGAGCGGGACTCTG), and the Cm-resistance marker of pSG76-CS (GeneBank: AF402780.1) was amplified with primers pSGCSH-FW (GC*TCTAGA*GTGAGGCACCAATAACTG) and pSGCSH-Rev (ACT*GTCGAC*GATCGGCACGTAAGAGGTTC). Both PCR products were double-digested with *Xba*I and *Sal*I, and were ligated using T4 ligase. Plasmid pMSD8 was a kind gift of Prof. John Cronan (University of Illinois, Urbana, IL, USA), and plasmids pETM6-M*accABCD* and pETM6-P*accABCD* (Xu et al., 2013) were kindly provided by Prof. Mattheos Koffas (Rensselaer Polytechnic Institute, Troy, NY, USA). The construction of plasmids pRSF*matCmatB*, pRSF*mmsA*, pRSF*cagg1256*, pRSFM*accABCD*, and pRSF*matCatoDA* was described elsewhere (Fehér et al., 2014). Plasmid pACYC*matCmatB* (Wu et al., 2013) was a kind gift of Dr. Jingwen Zhou (LBBE, Jiangnan, China). pACYCM*accABCD* was constructed by double-digesting pRSFM*accABCD* with *Bam*HI and *Avr*II, and ligating the 4855 bp-long fragment with the similarly digested pACYC*matCmatB*. pACYC*matCatoDA* was engineered by double-digesting pRSF*matCatoDA* with *Nde*I and *Xho*I, and ligating the 1408 bp fragment with the 5273 bp fragment of the similarly digested pACYC*matCmatB*. pACYC*mmsA* and pACYC*cagg1256* were constructed by double-digesting pRSF*mmsA* and pRSF*cagg1256*, respectively, both with *Eco*RI and *Xho*I, and ligating the respective 1685 and 3848 bp fragments with the similarly digested pACYC*matCmatB*. Restriction endonucleases and T4 ligase were obtained from Thermo Fisher Scientific (Waltham, MA, USA), Q5 DNA polymerase for PCR was from New England Biolabs.

### Fluorescence measurement of bacterial cultures

Each bacterial strain to be measured was grown overnight in mineral salts medium (Hall, 1998) supplemented with 0.2% glucose and the appropriate antibiotics. On the day of the measurement, the cultures were diluted 20-fold in fresh medium, and dispensed into clear-bottomed black-walled 96-well plates (Costar Ref. 3603, Corning, NY, USA). Growth and measurement of the cultures took place in a TECAN Infinite 500 fluorescent reader, shaken and incubated at 37°C. Optical density was determined at 600 nm. Fluorescence intensity was recorded using an excitation wavelength of 580 ± 10 nm, and an emission wavelength of 610 ± 5 nm, with a gain set to 40. Readings were taken every 12 min. Antibiotics were used at the following concentrations: ampicillin (Ap): 50 μg/ml, chloramphenicol (Cm): 25 μg/ml, kanamycin (Km): 30 μg/ml. Na-malonate was administered at 2 mg/ml, cerulenin was used at 20 μg/ml, and β-alanine at 3 mM end concentrations. For characterization of the sensors’ response to malonyl-CoA, IPTG was administered in a concentration-series of 0.01, 0.1, 0.3, 0.6, 1, and 10 mM. Arabinose was used in a log10 series from 10^−4^ to 10^−1^% during optimization and at 10^−2^% for comparison of alternate malonyl-CoA producer constructs. All chemicals were obtained from SIGMA (St. Louis, MO, USA).

### Identification of the dynamic model of the sensor

In order to determine the dynamic response of the sensor, we consider three approximate dynamic models.

#### Zero-pole model

This model considers that the dynamics between biomass *X*(*t*) and fluorescence *R*(*t*) can be approximately described by an integral time constant τ*_p_*, a derivative time constant τ*_z_*, and a gain *K*:

$$\tau_{p}\frac{\mathrm{dR}\left(t\right)}{\mathrm{dt}}=-R\left(t\right)+K\left(X\left(t\right)-\tau_{z}\frac{\mathrm{dX}\left(t\right)}{\mathrm{dt}}\right)$$

The transfer function between the input and the output expressed through the Laplace transform notation is:

$$\frac{R\left(s\right)}{X\left(s\right)}=K\frac{1-\tau_{z}s}{1+\tau_{p}s}$$

#### 1 zero-2 poles model

In this model, we consider an additional integral time constant τ*_p_*_2_ between the input *X*(*t*) and the output *R*(*t*), represented in the following equation through an auxiliary state variable *X*_1_(*t*):

$$\begin{array}{llll} \tau_{p1}\frac{dX_{1}\left(t\right)}{\mathrm{dt}} & =-X_{1}\left(t\right)+K\left(X\left(t\right)-\tau_{z}\frac{\mathrm{dX}\left(t\right)}{\mathrm{dt}}\right)\; & & \;\\ \tau_{p2}\frac{\mathrm{dR}\left(t\right)}{\mathrm{dt}} & =-R\left(t\right)+X_{1}\left(t\right)\; & & \; \end{array}$$

In Laplace transform notation:

$$\frac{R\left(s\right)}{X\left(s\right)}=K\frac{1-\tau_{z}s}{\left(1+\tau_{p1}s\right)\left(1+\tau_{p2}s\right)}$$

#### 1 zero-3 poles model

In this model, we consider an additional integral time constant τ*_p_*_3_ between the input *X*(*t*) and the output *R*(*t*), represented in the following equation through an additional state variable *X*_2_(*t*):

$$\begin{array}{llll} \tau_{p1}\frac{dX_{1}\left(t\right)}{\mathrm{dt}} & =-X_{1}\left(t\right)+K\left(X\left(t\right)-\tau_{z}\frac{\mathrm{dX}\left(t\right)}{\mathrm{dt}}\right)\; & & \;\\ \tau_{p2}\frac{dX_{2}\left(t\right)}{\mathrm{dt}} & =-X_{2}\left(t\right)+X_{1}\left(t\right)\; & & \;\\ \tau_{p3}\frac{\mathrm{dR}\left(t\right)}{\mathrm{dt}} & =-R\left(t\right)+X_{2}\left(t\right)\; & & \; \end{array}$$

In Laplace transform notation:

$$\frac{R\left(s\right)}{X\left(s\right)}=K\frac{1-\tau_{z}s}{\left(1+\tau_{p1}s\right)\left(1+\tau_{p2}s\right)\left(1+\tau_{p3}s\right)}$$

In order to identify the model from the samples, we used an approximate discrete model based on the bilinear transform (Oppenheim and Schafer, 2010):

$$s\leftarrow \frac{2}{T_{s}}\frac{z-1}{z+1}$$

where *z*^−1^ corresponds to a pure delay in the sample and *T*_s_ is the sampling frequency. Parameters of the discrete model were fitted by linear regression using the R package with the dynlm package (Zeileis, 2014), which was also employed to perform the simulations.

## Results

### Experimental assessment of malonyl-CoA sensing

Malonyl-CoA is an important building block from the aspect of metabolic engineering, for it is required for the biosynthesis of fatty acids, polyketides, flavonoids, and other compounds (Fowler et al., 2009; Xu et al., 2011). In an earlier work, we have predicted and tested several alternative pathways that yield malonyl-CoA in order to boost pinocembrin production in *E. coli* (Fehér et al., 2014). Briefly, one candidate for malonyl-CoA synthase (*matB*, EC 6.2.1), one for malonate-CoA transferase (*atoDA*, EC 2.8.3.3), two for malonyl-CoA reductase (*mmsA* and *cagg1256*, EC 1.2.1.75) as well as one for the acetyl-CoA carboxylase complex (*accABCD* from *E. coli*, EC 6.4.1.2) were expressed together with the complete pinocembrin pathway, and their efficiencies were deduced from the resulting normalized pinocembrin titers. Here, we used the molecular sensor as a more direct method to investigate the levels of malonyl-CoA produced by the alternative enzymes.

#### Verifying the function of the malonyl-CoA sensor

**Figure 1:**
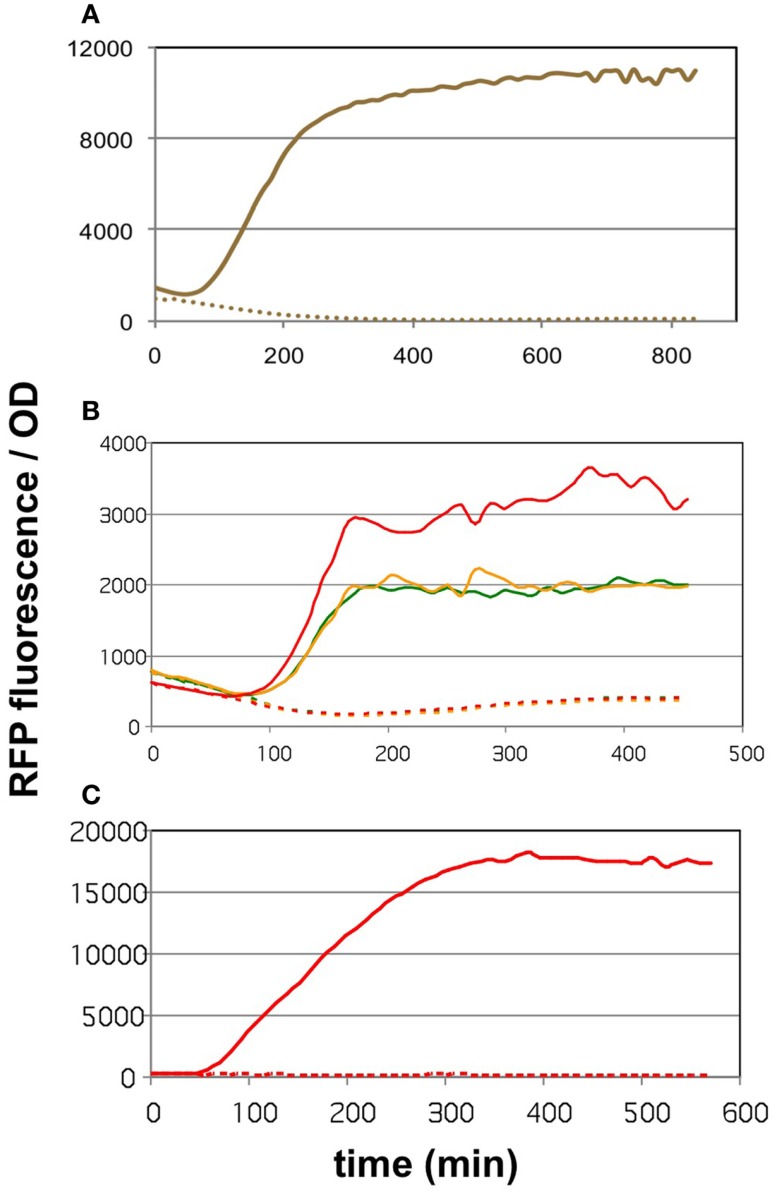
**The effect of cerulenin on fluorescence generated by the sensor circuits**. On all three graphs, the solid lines show the cultures challenged with cerulenin, while the segmented lines depict the corresponding untreated cultures. **(A)** DH5α cells harboring the pBFR1k_RFP_8FapR sensor plasmid in mineral salts medium. **(B)** DH5α cells harboring the pCFR sensor plasmid in LB medium. Three cultures were tested, originating from three colonies obtained after transformation of the pCFR ligate. **(C)** DH5α cells harboring the pCFR sensor plasmid from the best performer colony seen in **(B)**, cultured in mineral salts medium.

The core of the malonyl-CoA sensor is an RFP gene driven by the synthetic pFR1 promoter (Liu et al., 2015). pFR1 is a combination of P_A1_ promoter from phage T7 (Deuschle et al., 1986) and two FapR-binding segments flanking the −10 region. The FapR protein, originating from *Bacillus subtilis*, dissociates from the DNA upon the binding of malonyl-CoA (Schujman et al., 2003), thereby allowing the *E. coli* RNA polymerase to transcribe the downstream sequences. The transcription of FapR is driven by a pAra promoter (controlled by the AraC protein), pFR1 is therefore increasingly repressible by elevating the l-arabinose levels. The AraC and FapR transcription factors, as well as RFP are all encoded on pBFR1k_RFP_8FapR, a kanamycin-resistant plasmid constructed by the workgroup of Fuzhong Zhang (Liu et al., 2015). The authors validated the quantitative nature of malonyl-CoA sensing by generating a calibration curve that correlated the fluorescence levels to LC-MS measurements of intracellular malonyl-CoA. In order to use this sensor to compare the malonyl-CoA productivity of our construct collection available on the kanamycin-resistant pRSF vector, we generated a chloramphenicol-resistant version of the plasmid, which we call pCFR (plasmids used in this study are listed in Table S1 in Supplementary Material). The functionality of pCFR, as well as that of the original sensor plasmid was benchmarked with the help of cerulenin. This is an inhibitor of 3-ketoacyl-ACP synthase I and II (D’Agnolo et al., 1973), which participate in the committing step of free fatty acid biosynthesis. The inhibition of these enzymes results in a substantial intracellular accumulation of malonyl-CoA (Davis et al., 2000), and should therefore cause an increase in RFP fluorescence. Indeed, *E. coli* cells carrying either pBFR1k_RFP_8FapR or pCFR responded to cerulenin with a sharp increase in OD-normalized fluorescence (Figures 1A,B). As a matter of fact, a small scale screening of four pCFR-carrying clones allowed the identification of the most effectively functioning plasmid, which was chosen for all downstream experiments (Figure 1B). As expected, the ratio of RFP/OD values of cerulenin-treated to control cultures was higher in minimal medium than in LB broth (Figure 1C), due to the relatively high background-fluorescence of LB medium (Xu et al., 1999). For this reason, all further measurements were carried out in minimal medium.

#### Optimizing sensitivity toward malonyl-CoA detection

**Figure 2:**
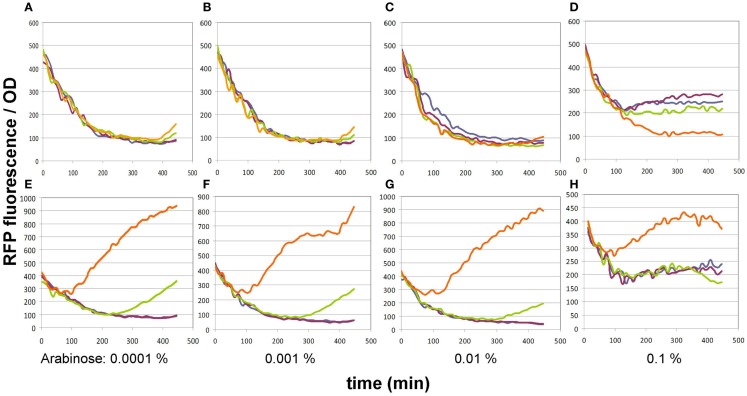
**Fluorescence generated by the pBFR1k_RFP_8FapR sensor plasmid in response to various arabinose and IPTG concentrations**. **(A–D)** depict control BL21DE3 cells harboring only the pBFR1k_RFP_8FapR plasmid, while **(E–H)** show BL21DE3 cells carrying the pBFR1k_RFP_8FapR and the pACYC*matCmatB* plasmids. Arabinose concentrations are shown under each pair of graphs. IPTG concentrations are as follows: blue: 0.001 mM; purple: 0.01 mM; green: 0.1 mM; orange: 1 mM. Na-malonate was included in the medium for all cultures.

In its original publication by Liu et al. (2015), the malonyl-CoA sensor was characterized in a split form: the FapR and RFP genes were carried by two separate plasmids. Therefore, we re-characterized pBFR1k_RFP_8FapR in order to find the optimal conditions of comparing various malonyl-CoA producer enzymes using the single-plasmid sensor construct. The expression level of the FapR repressor was varied by administering a dilution series of arabinose, while the expression level of the malonate transporter (*matC*) and the *matB* from plasmid pACYC*matCmatB* was controlled by altering the IPTG concentration. A 4 × 4 concentration matrix was thus applied to cells carrying either both plasmids or just the sensor plasmid. As apparent from the OD-normalized fluorescence values shown on Figure 2, an arabinose concentration of 0.01% proved to be optimal, taking both specificity and sensitivity into account, similarly to the originally published sensor system. At lower arabinose concentrations, specificity deteriorated, since the sensor plasmid produced slight increases in fluorescence in response to IPTG even in the absence of the malonyl-CoA producer plasmid. This could indicate the promoter’s non-specific binding to LacI, which is competitively inhibited by sufficient quantities of the FapR protein. Increasing arabinose levels to 0.1% strongly reduced fluorescence levels of the sample, leading to a decrease of sensitivity and of the signal to noise ratio, possibly resulting from an over-repression by FapR. The initial decrease in fluorescence/OD values, seen on all graphs is likely due to the delay of RFP expression compared to culture growth.

#### Comparison of malonyl-CoA-producing construct collection

**Figure 3:**
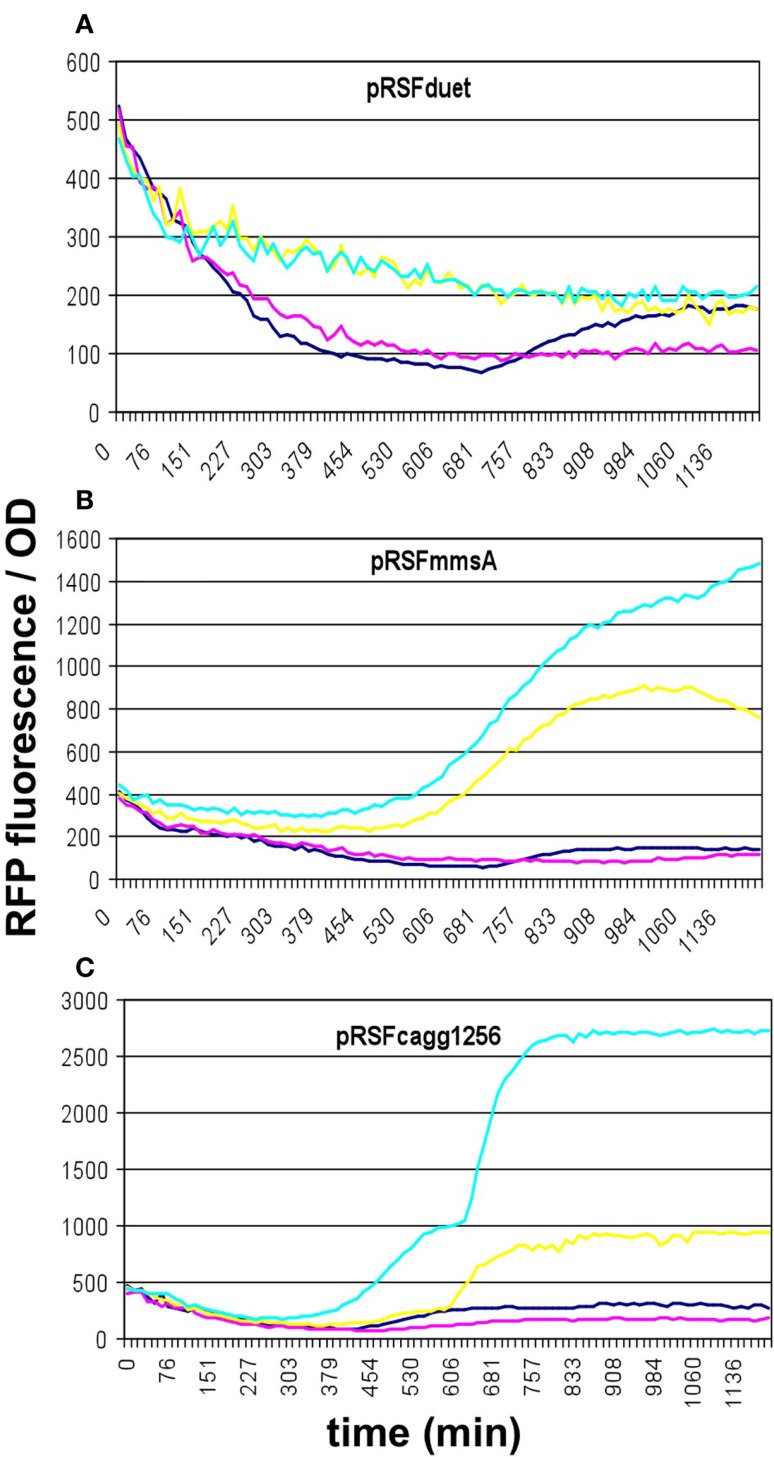
**Fluorescence response of BL21DE3 cells carrying pCFR sensor plasmid and (A) the empty pRSFduet vector, (B) pRSF*mmsA*, or (C) pRSF*cagg1256***. For **(B,C)**, β-alanine was included in the medium. Colors indicate the concentration of IPTG used for induction: dark blue: 0.01 mM; magenta: 0.01 mM; yellow: 1 mM; cyan: 10 mM.

**Figure 4:**
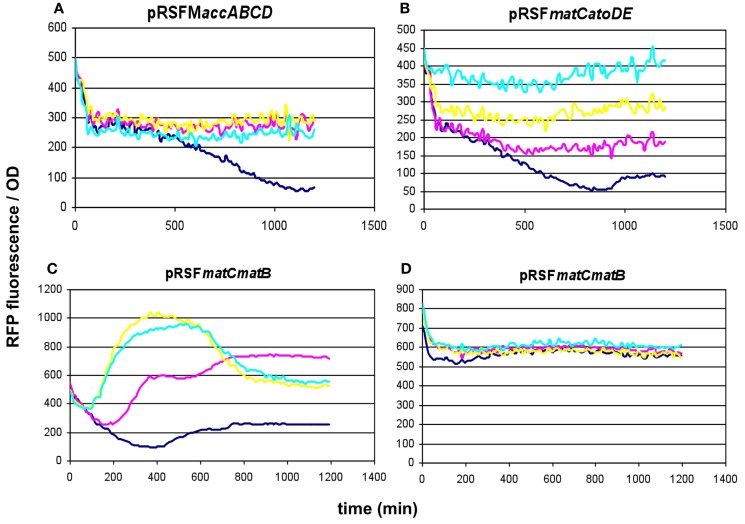
**Fluorescence response of BL21DE3 cells carrying pCFR sensor plasmid and (A) pRSFM*accABCD* or (B) pRSF*matCatoDA***. **(C,D)** show two distinct fluorescence responses to IPTG induction of cells carrying pRSF*matCmatB*. Na-malonate was included in the medium for **(B–D)**. Colors indicate the concentration of IPTG used for induction: dark blue: 0.01 mM; magenta: 0.1 mM; yellow: 1 mM; cyan: 10 mM.

To compare the malonyl-CoA production efficiencies of alternative malonyl-CoA-producing pathways, at least one representative enzyme for each pathway was cloned, as described earlier (Fehér et al., 2014). Briefly, the *matB*, *atoDA*, *mmsA*, and *cagg1256* genes, as well as the *accABCD* gene complex were expressed from the high-copy plasmid pRSFduet. To provide sufficient amounts of substrate, a MatC was co-expressed with *matB* and *atoDA*, and the medium was supplemented with Na-malonate. To elevate the substrate levels for *mmsA* and *cagg1256*, cultures were supplemented with β-alanine, which is converted to 3-oxopropanoate (malonate semialdehyde) by the cell’s endogenous 4-aminobutyrate aminotransferase. In every case, the fluorescence/OD values were monitored over time after growing the cells in various IPTG concentrations. Cells carrying the empty pRSFduet plasmid (besides the sensor plasmid) were used as a negative control. Based on their response, the strains carrying the various constructs fell into either one of the two following categories: (i) the cells exhibited growth and a dose-dependent fluorescence/OD response to IPTG (pRSF*mmsA* and pRSF*cagg*) (Figure 3) or (ii) the cells exhibited absolutely no growth upon IPTG induction in minimal medium (pRSFM*accABCD* and pRSF*atoDA*) (Figures 4A,B). It is important to note that certain cells of the latter category also gave a seemingly dose-dependent response to IPTG (such as pRSF*atoDA*), but their fluorescence and OD values were practically unchanged during the course of the experiment, most probably indicating that the trend in their ratio is an artifact (Figure S1 in Supplementary Material). Interestingly, both types of responses were seen with pRSF*matCmatB*-carrying cells, in a quite irreproducible manner (see Discussion and Figures 4C,D and Figure S2 in Supplementary Material). The growth curves obtained by inducing strains carrying various producer plasmids are summarized on Figure S3 in Supplementary Material.

#### Investigating lower-copy-number alternative constructs

**Figure 5:**
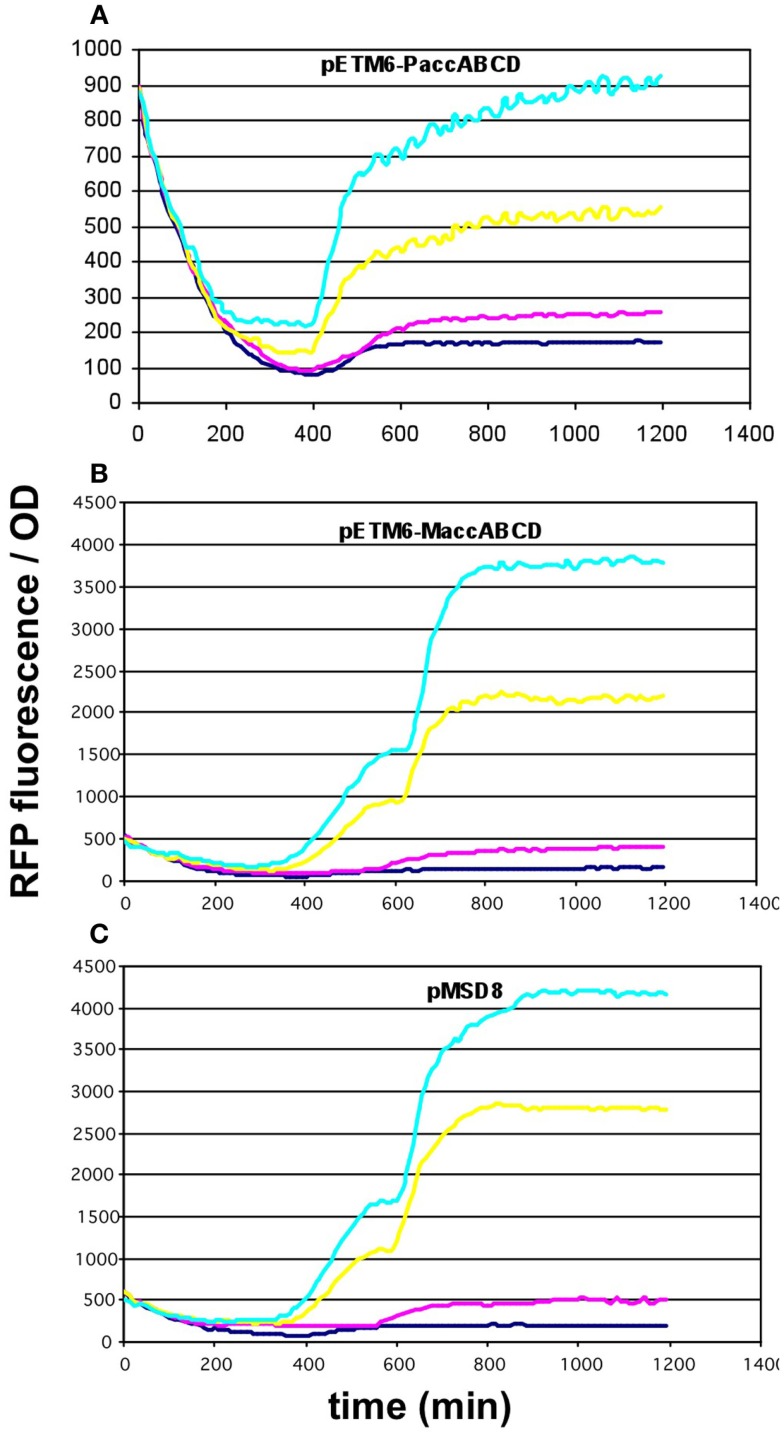
**Fluorescence response of BL21DE3 cells carrying pCFR sensor plasmid and (A) pETM6-P*accABCD*, (B) pETM6-M*accABCD*, or (C) pMSD8**. Colors indicate the concentration of IPTG used for induction: dark blue: 0.01 mM; magenta: 0.1 mM; yellow: 1 mM; cyan: 10 mM.

Since the overexpression of certain members of the *accABCD* complex (Davis et al., 2000), as well as protein overproduction in general (Flores et al., 2004) have been described to have a toxic effect on the cells, the overexpression of certain constructs was repeated using lower-copy vectors. For the *accABCD* complex, pMSD8, an ultra-low-copy variant was available expressing the four genes as a T7-driven operon. In addition, two medium-copy alternatives, pETM6-P*accABCD* and pETM6-M*accABCD*, carrying the genes as a pseudo-operon (separate promoters) and in monocistronic form (separate promoters and terminators), respectively, were also obtained and tested (Xu et al., 2012, 2013). All three plasmids displayed a clear dose-dependent fluorescence/OD pattern, well above the levels of the negative control (Figures 5A–C). For *matCmatB*, pACYC*matCmatB* was readily available as a low-copy alternative that functioned in a quite well-reproducible fashion (Figure 2), when tested with the pBFR1k_RFP_8FapR sensor plasmid.

**Figure 6:**
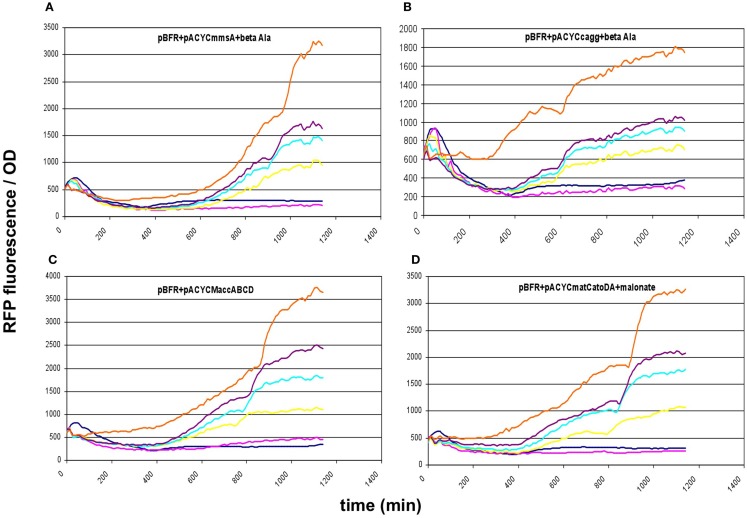
**Fluorescence response of BL21DE3 cells carrying pBFR1k_RFP_8FapR sensor plasmid and (A) pACYC*mmsA*, (B) pACYC*cagg1256*, (C) pACYCM*accABCD*, or (D) pACYC*matCatoDA***. The medium was supplemented with β-alanine for pACYC*mmsA* and pACYC*cagg1256*, and with Na-malonate for pACYC*matCatoDA*. Colors indicate the concentration of IPTG used for induction: dark blue: 0.01 mM; magenta: 0.1 mM; yellow: 0.3 mM; cyan: 0.6 mM; purple: 1 mM; orange: 10 mM.

These results prompted us to subclone our pathway collection into a pACYC backbone, thereby reducing the copy-number of all constructs to ~15. When expressed this way, all five constructs (*matCmatB*, *mmsA*, *cagg1256*, *M.accABCD*, and *matCatoDA*) allowed cell growth and displayed a scalable, IPTG-dependent fluorescence (Figures 2 and 6). As it turned out, the concise nature of the dataset obtained with the pACYC-collection allowed us to fit various models describing the response of the sensor to malonyl-CoA production (see below).

#### Quantification and reproducibility of malonyl-CoA levels

Since *E. coli* strain BL21DE3, the host used in our experiments is not deleted for the genes of arabinose catabolism, it breaks down arabinose when the medium is depleted for glucose. This likely causes a decrease in FapR expression, and a consequent derepression of RFP at the end of growth. As a result, a second, sudden increase in fluorescence was seen in many samples upon the transition to stationary phase, as apparent from juxtaposing the temporal evolution of fluorescence and OD (Figure S4 in Supplementary Material). To avoid this effect, we analyzed fluorescence levels corresponding to the log-phase (OD = 0.6), a physiological state when arabinose catabolism is still inhibited by the catabolite repression of *E. coli*. Figure S5 in Supplementary Material shows some of the fluorescence vs. OD plots generated to obtain such readings.

**Figure 7:**
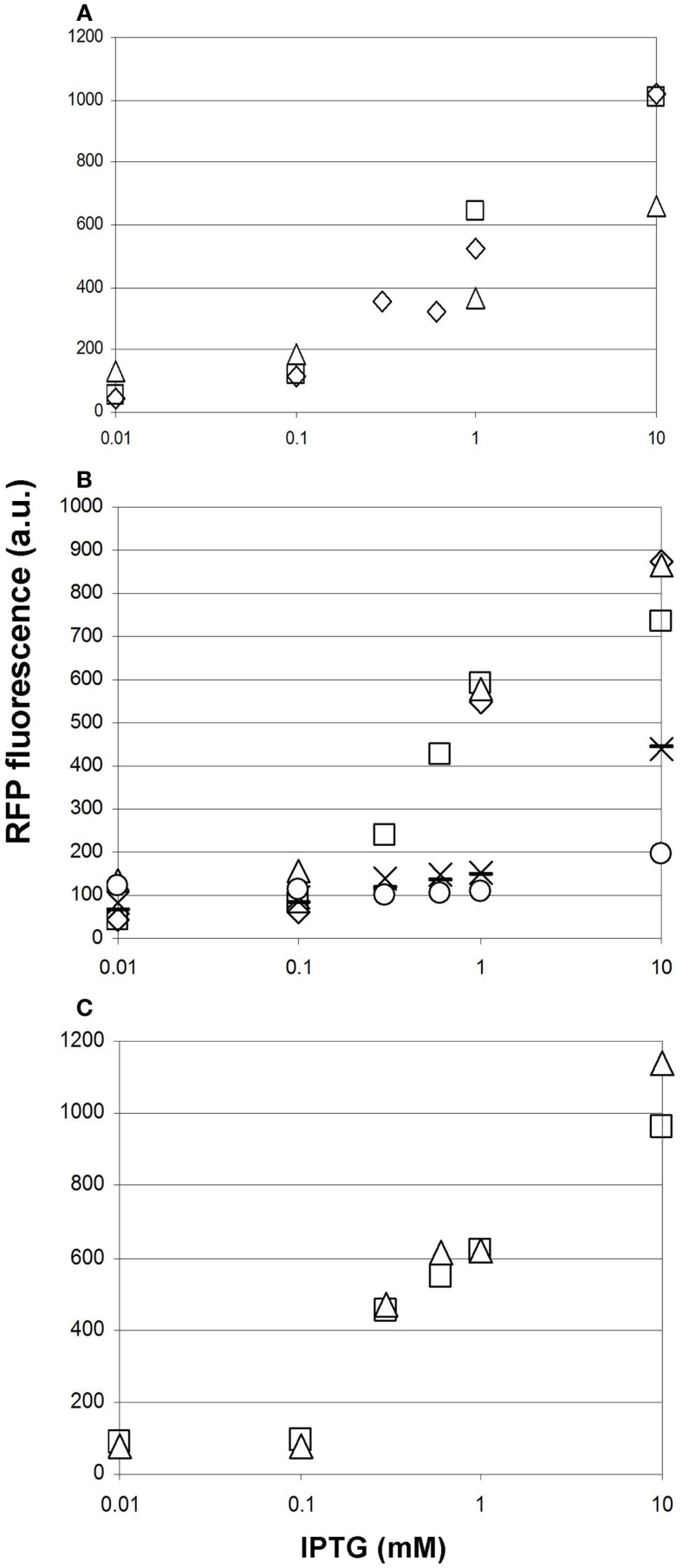
**Reproducibility of measurements of malonyl-CoA production in BL21DE3 cells using the fluorescent sensor plasmid pCFR and various production plasmids**. The IPTG-dependence of fluorescence measured at OD = 0.6 is shown in every case. **(A)** Two cultures harboring pMSD8, originating from two distinct colonies after transformation are depicted by open squares and open triangles. Open diamonds represent the re-measurement of the strain corresponding to the open squares, 1 day later. **(B)** Six cultures harboring ETM6-M*accABCD* measured on different days. **(C)** Two cultures of the same clone of pACYC*matCmatB* originating from the same starter culture, measured in the same run, in the presence of Na-malonate.

Prior to comparing the performance of various malonyl-CoA-producing constructs, we investigated the reproducibility of our measurements at three different levels. First, we compared the fluorescence values of parallel clones, resulting from the transformation of a given producer construct. These displayed a strong variation in their response to IPTG, which was not unexpected taking into account their heterologous colony morphologies (Figure 7A). Second, we re-tested the best performing clones after their storage in the form of glycerol stocks. Reproducibility was substantially better, being the best for the lowest copy-number pMSD8 plasmid (Figure 7A). For other constructs, the performance seemed to fall into two distinct clusters: it either matched that of earlier measurements, or deteriorated to a lower, near-zero value, as demonstrated by the dose–response curves of pETM6-M*accABCD* (Figure 7B). This phenomenon was reminiscent of a burden-relieving mutation that swept through the population early after induction. The third level of reproducibility corresponds to comparing the performance of a given strain in the same microtiter plate in the same measurement-run, starting from the same overnight seed-culture (Figure 7C). The high reproducibility of such measurements was sufficient to draw conclusions on the effect of providing the substrates for malonyl-CoA production (see below).

#### Investigating the effect of administering the substrate

**Figure 8:**
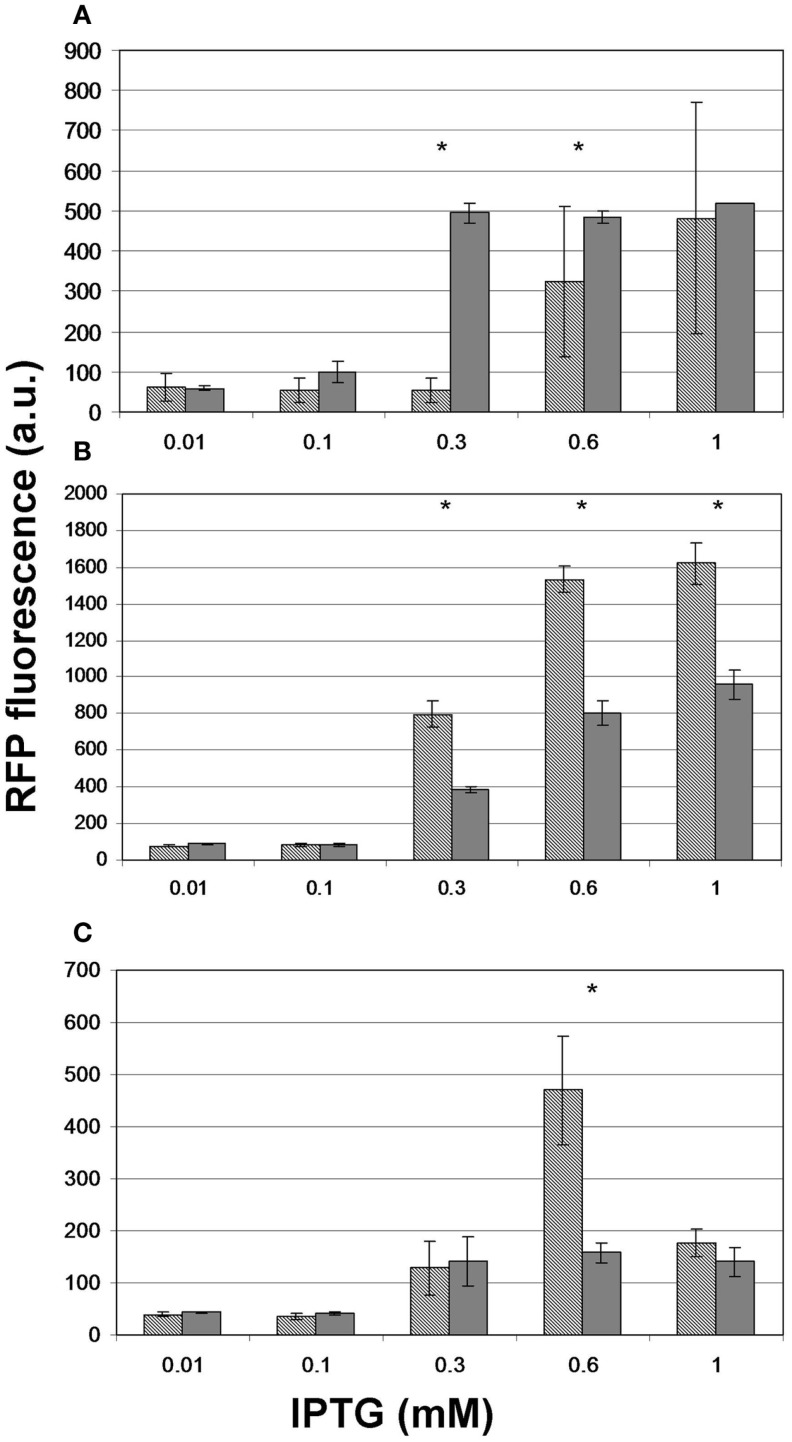
**The effect of administering the substrate on the level of fluorescence obtained upon induction of malonyl-CoA production**. Hashed bars represent the effect of IPTG on the fluorescence obtained at OD = 0.6 with constructs **(A)** pACYC*matCmatB*, **(B)** pACYC*matCatoDA*, and **(C)** pRSF*mmsA*. Gray bars depict the peak fluorescence detected when inducing the same constructs in the presence of their substrate: Na-malonate for *matCmatB* and *matCatoDA* and β-alanine for *mmsA*. The sensor plasmid was pBFR1k_RFP_8FapR for **(A,B)** and pCFR for **(C)**. Asterisks indicate a significantly different fluorescence caused by the substrate (*p* < 0.05).

To test whether providing the substrates of malonyl-CoA synthesis in the growth medium offers a measurable advantage in the product levels, we repeated the experiment for, pACYC*matCmatB* and pACYC*matCatoDA* with and without Na-malonate as well as for pRSF*mmsA* and pRSF*cagg* with and without β-alanine. For pACYC*matCmatB*, we observed significantly boosted production at 0.3 and 0.6 mM IPTG (Figure 8A). However, the presence of Na-malonate significantly hindered the performance of pACYC*matCatoDA* at IPTG concentrations of 0.3 mM and above (Figure 8B). Similarly, supplementing β-alanine to pRSF*mmsA* seemed to have a negative effect at 0.6 mM IPTG induction (Figure 8C). No significant effect was seen for adding the substrate to pRSF*cagg* (not shown).

#### Investigating the efficiencies of malonyl-CoA production

The four alternative pathways for malonyl-CoA production, as well as the top-scoring enzymes that catalyze the required steps were sought for by RetroPath, our retrosynthetic biology tool developed for metabolic engineering (Carbonell et al., 2011). As opposed to our initial plans, the growth-inhibitory effect of certain pRSF-based constructs prohibited us to validate the ranking of the alternative pathways by direct comparison of their measured fluorescence values. Indeed, the toxicity of overproducing malonyl-CoA by overexpression of acetyl-CoA carboxylase has been described before (Davis et al., 2000). The performance of the readily available low-copy variants of *accABCD*, however (pMSD8, pETM6-P*accABCD*, and pETM6-M*accABCD*), indicated the importance of using lower-copy expression vectors. After recloning our gene-set into pACYC-derived plasmids, *matCatoDA*, *MaccABCD*, *mmsA*, *cagg1256*, and *matCmatB* all permitted growth when expressed. Importantly, based on their fluorescence values, these constructs all produced significantly more malonyl-CoA when induced with 1 mM of IPTG, than the control cell harboring no production (see below). Ranking the alternative malonyl-CoA-producing pathways was not feasible due to the high variation of the data. Important practical conclusions could be nevertheless made, listed in the Section “Discussion.”

### Modeling the malonyl-CoA sensor

#### Characterization of the sensor response

In order to characterize the sensor, we modeled the sensor response to the production of malonyl-CoA, its measured signal. Ideally, the sensor once calibrated should display a good reproducibility as well as a stable sensitivity for its use in applications such as screening or feedback regulation. For that purpose, it is desirable to keep the dynamic response of the sensor uncoupled from any parameter variation so that the relationship with the measured signal and its output corresponds basically to a linear one. The time of response of such linear sensor, on the other hand, should be kept below some reasonable limit in order to avoid large lags introducing potential instability into the system. Here, as we are basing our model on the observations of OD and RFP, we need to consider in our model the relation between these variables with respect to the malonyl-CoA concentration, the signal that is measured by the sensor. In this study, the model necessarily will be kept simple, as our purpose is to provide a set of parameters that can be easily measured in order to calibrate the sensor.

**Figure 9:**
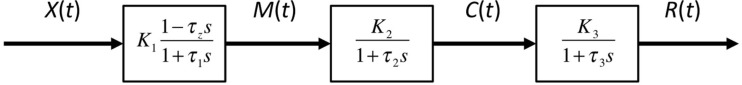
**An approximate model of the response behavior of the sensor based on a cascade of first-order filters**. The biomass *X*(*t*) is assumed to be approximately related to the concentration of malonyl-CoA *M*(*t*) through a first-order pole-zero filter. The sensor complex malonyl-CoA-FapR concentration *C*(*t*) is then related to *M*(*t*) through a first-order filter and finally, the RFP concentration *R*(*t*) is again related to *C*(*t*) through another first-order filter.

Similarly to other proposed models (Anesiadis et al., 2008), we consider that the signal undergoes several transformation stages in cascade-mode going from the biomass to the fluorescence levels:

1. We assume that the variation on concentration of malonyl-CoA *M(t)* depends on its instant production, given by the rate *v*_m_, which is specific to each selected malonyl-CoA-producing construct or pathway, multiplied by the biomass *X*(*t*) minus the malonyl-CoA that is consumed for growth defined as a constant rate *v*_g_ that multiplies the variation in time of biomass and another term for degradation, defined by a rate constant γ_m_:
  (1) $$\frac{\mathrm{dM}\left(t\right)}{\mathrm{dt}}=v_{m}X\left(t\right)-v_{g}\frac{\mathrm{dX}\left(t\right)}{\mathrm{dt}}-\gamma_{m}M\left(t\right)$$
  During the exponential growth phase, this previous equation is simplified as:
  (2) $$\frac{\mathrm{dM}\left(t\right)}{\mathrm{dt}}=\left(v_{m}-\mu v_{g}\right)X\left(t\right)-\gamma_{m}M\left(t\right)$$
  where μ is the specific growth rate.
2. Malonyl-CoA binds to transcription factor FapR to form a complex whose concentration *C*(*t*) depends on a dissociation constant *K*_d_ [2.4 μM according to Schujman et al. (2006)]. The concentration of FapR *F*(*t*) is considered constant depending on the concentration levels of the inducer l-arabinose:
  (3) $$\begin{array}{llll} & F+M\overset{k_{f}}{\underset{k_{r}}{⇌}}C\; & & \;\\ & \frac{\mathrm{dC}\left(t\right)}{\mathrm{dt}}=k_{f}\mathrm{FM}\left(t\right)-k_{r}C\left(t\right) \end{array}$$
3. The change on concentration of RFP *R*(*t*), depends on the concentration of the complex *C*(*t*) through a constant κ associated to the promoter strength (Oyarzún and Stan, 2013) and a decay constant γ_r_:
  (4) $$\frac{\mathrm{dR}\left(t\right)}{\mathrm{dt}}=\kappa C\left(t\right)-\gamma_{r}R\left(t\right)$$
  Under the assumptions given by the model described by Eqs 1–4, the response of the sensor can be approximated as a cascade of filters as shown in Figure 9. The advantage of using such approximation is that we can assume that the dynamics due to the time constants of these three filters occur at different time scales and therefore, under the appropriate conditions, time-scale separations can be performed. Based on this principle, we can approximate the model by considering main time constants present in the model, i.e., by estimating parameters of a cascade of first-order models (see Materials and Methods).

#### Sensor model fitting

In our sensor model, we are assuming that under same inducer and regulatory conditions on a strain population, malonyl-CoA levels should change basically because of the change in the rate of production of this metabolite by the strain (v_m_ in Eq. 1), which would correspond to a different gain of the sensor, i.e., a different measured RFP and biomass *X* in steady state.

Such gain according to the sensor model in Eqs 1–4, the gain or relationship in steady state between RFP and *X* is given by the following relationship between model parameters:

(5) $$\frac{R}{X}=K_{d}\frac{v_{m}}{\gamma_{m}}\frac{\kappa }{\gamma_{r}}$$

Regarding model dynamics, we considered three simplified models for the response dynamics depending on the time constants that are considered (see Materials and Methods): (1) a first-order with integral and derivative terms; (2) a second-order model consisting of a first-order model with integral and derivative terms and an additional first-order integral term; and (3) a third-order model with the integral and derivative terms and two additional first-order integral terms.

**Figure 10:**
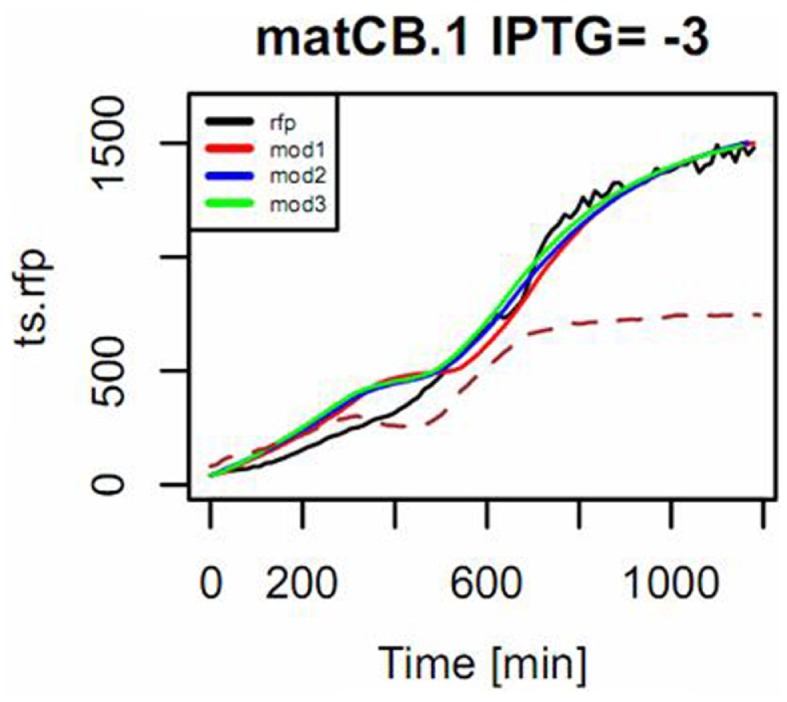
**Example of the mode of fitting models to describe the response of the pBFR1k_RFP_8FapR sensor in the presence of pACYC*matCmatB*, induced with 1 mM of IPTG**. Na-malonate was included in the medium. The input signal is the measured OD (shown in dotted line and scaled to 50% of maximum in the plot) and the simulated response of the model for the observed output RFP (in black) is shown for a fitting to a first-order model with input derivative (red), a second-order with input derivative (blue), and a third-order with input derivative (green).

As shown in Figure 10, measured responses were in general fitted with an increasing level of accuracy to each of the three approximate sensor models. For some constructs, however, measurements could not be successfully fitted to model 3 (see Figure S4 in Supplementary Material). Therefore, we decided to use model 2 (the one considering one derivative and two integral terms) as the reference model in order to characterize the dynamics of the sensor. Table 1 provides a list of estimated parameters for different constructs. Time constants ranged between approximately 1 and 10 h.

**Table 1:** **Gain and integral time constants of the sensor fitted to model 2 estimated for different constructs for IPTG = 1 × 10^−5^**.

Construct	*K*	τ_1_	τ_2_
matCmatB	250.84	290.11	12.69
mmsA	300.53	288.31	15.67
cagg	362.82	525.90	17.39
acc	320.20	417.92	11.49
matCatoDA	321.12	156.95	11.14

#### Dependence of sensor parameters on induction

**Figure 11:**
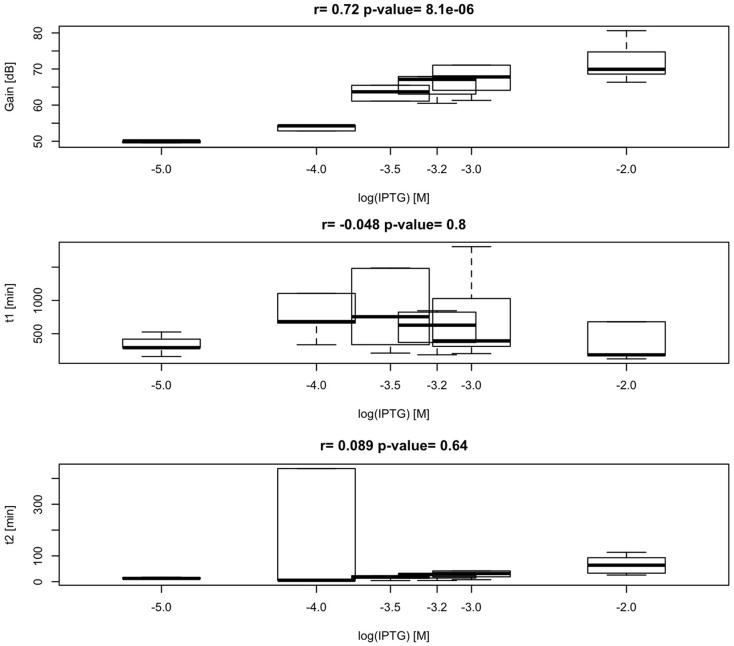
**Variability and correlation between model parameters *K*, τ_p_, τ_z_ of the sensor, and IPTG levels 0.01, 0.1, 0.3, 0.6, 1, and 10 mM**. Calculated correlation between parameters *K*, τ_p_, τ_z_, and IPTG levels are shown.

**Figure 12:**
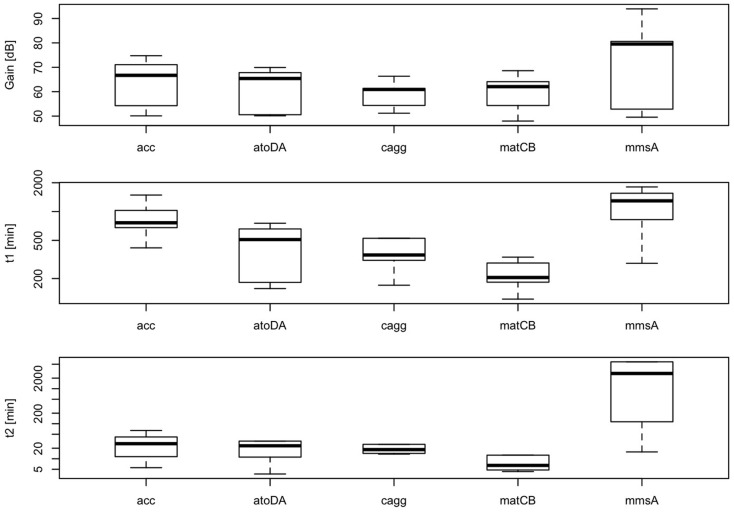
**Variability of parameters *K*, τ_p_, τ_z_ of the sensor for each construct at several IPTG concentrations (0.01, 0.1, 0.3, 0.6, 1, and 10 mM)**. Calculated correlation between parameters *K*, τ_p_, τ_z_, and IPTG levels are shown.

According to our simplified model of the sensor consisting on a second-order transfer function, gain between biomass and RFP should depend on the level of induction of the malonyl-CoA-producing enzyme. Time constants, on the contrary, should not depend on the level of induction, but should be constitutive parameters of the sensor that can be calibrated for each construct. As shown in Figure 11, we found effectively a good correlation between IPTG levels and sensor gain (*r* = 0.72, *p*-value = 8.1 × 10^−6^), indicating that the sensor is responsive to changes in induction of the enzyme. Time constants, as expected, were not significantly affected by changes in the IPTG concentration. These time constants, on the other hand, appear as distinctively clustered for each construct, showing the robustness of the sensor (Figure 12).

## Discussion

**Figure 13:**
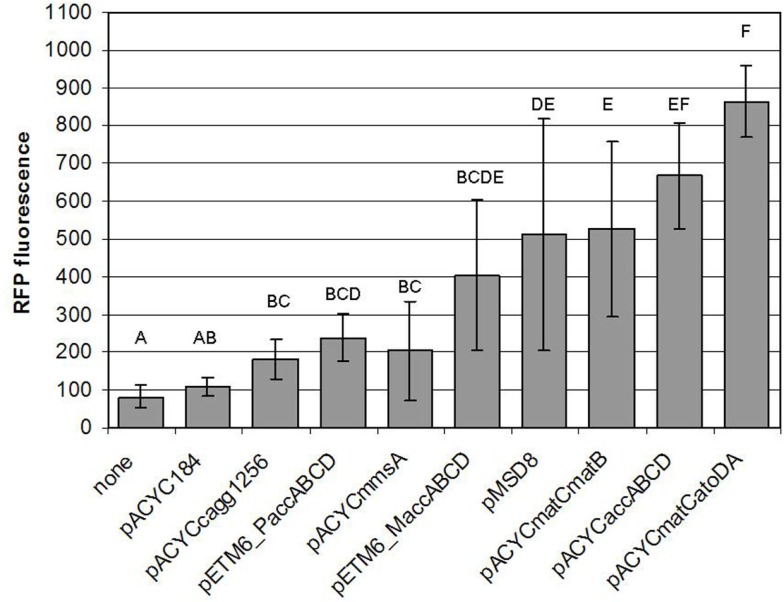
**Comparison of the malonyl-CoA levels produced by various constructs**. Fluorescence observed at OD = 0.6, generated by pBFR1k_RFP_8FapR upon induction of the corresponding producer plasmid with 1 mM IPTG in a BL21DE3 host is depicted. Na-malonate was added for pACYC*matCmatB* and pACYC*matCatoDA*, and β-alanine was administered for pACYC*mmsA* and pACYC*cagg1256*. Each letter represents a value significantly differing from the others [i.e., if two value were found to significantly differ from each other (*p* < 0.05), then the letters corresponding to their bars do not overlap].

In this work, we have successfully implemented a malonyl-CoA sensor for the partial optimization of malonyl-CoA production in *E. coli* BL21DE3. Initially, we screened several conditions to be used for the measurements, and found the optimal one to match that described earlier (Liu et al., 2015), despite the possible changes in relative gene dosage caused by the single-plasmid vs. dual-plasmid layout. We constructed a chloramphenicol-resistant version of the plasmid as well, to compare our construct collection encoding four alternative pathways for malonyl-CoA production, predicted by RetroPath. Besides their comparison, our aim was to use malonyl-CoA sensing to seek optimal conditions for efficient malonyl-CoA production, previously seen to be hindered by growth retardation (Fehér et al., 2014). Our current results also indicated, that the viability of the cells can be, in many cases strongly impaired by the high expression levels of the original pRSF-based constructs (Figure S3 in Supplementary Material). It was also apparent, that the lower-copy alternatives available for the *accABCD* gene complex (pMSD8, pETM6-M*accABCD*, and pETM6-P*accABCD*) outperformed all or most members of the RSF-collection. Therefore, a partial optimization of expression levels was carried out by using smaller copy-number vectors to obtain viable cells with measurable malonyl-CoA production upon induction, leading to a successful circumvention of the possible toxicity caused by the high-copy expression vectors. Due to the relatively high variation of the resulting data, comparison of these constructs was not conclusive enough to validate all our predicted rankings. However, the obtained measurements were quite useful for the practical purpose of selecting the most efficient implementation of our malonyl-CoA-producing constructs. From this aspect, the best performer of our construct set (in terms of fluorescence at OD = 0.6) turned out to be pACYC*matCatoDA* carrying the malonate-CoA transferase on a P15a-derived low-copy-number plasmid (Figure 13). Its performance could be nonetheless matched by the expression of the acetyl-CoA carboxylase complex, which, in turn was indiscernible from the *matB*. The two genes encoding malonyl-CoA reductase (*mmsA* and *cagg1256*) performed significantly weaker. Importantly, the activity of these constructs uncovered two pathways, malonyl-CoA reductase and malonate-CoA transferase, which have not been implemented before in boosting malonyl-CoA biosynthesis.

When comparing the variation of measured fluorescence values, we observed a consistent decrease in the coefficient of variation for all constructs when recloned into the low-copy pACYC vector, possibly marking an increased genetic stability (Table S2 in Supplementary Material). In theory, this change could also be caused by the fact of using different sensor plasmids in the comparison. However, no such difference was seen in the case of the pET-based plasmids when switching from pCFR to pBFR1k_RFP_8FapR used for sensing (Table S2 in Supplementary Material), indicating that it is the change in copy-number of the malonyl-CoA producer plasmid, and not the change in the sensor plasmid that caused the decrease of variation. This variation can be, at least in part, explained by burden-relieving mutations that sweep through the population early after induction. In fact, such an expansion of a non-fluorescent subpopulation was seen real time during one of our tests concerning pRSF*matCmatB* (Figure 4C), which was the least reproducible among all of our constructs. In that specific experiment, the fluorescence of the population ceased to grow after a certain point of time, despite the accelerating increase of the OD, leading to a decrease of the fluorescence/OD ratio (Figure S2 in Supplementary Material). The fact that no such effect is seen in Figure 4D indicates the random and incidental nature of this phenomenon, supporting the assumption that it is caused by a mutation. The specific mutations inactivating the production were not sought for.

In their original publication, the designers of this sensor detected a saturation of malonyl-CoA levels at 25 μM, when inducing the expression of the *acc* operon at different strengths (Liu et al., 2015). This phenomenon was not investigated further, but could have been caused by the toxicity of the produced compound. Although we did not observe an unambiguous plateau of fluorescence values in our experiments, the possibility of such a scenario should be taken into account when using similar systems in the future. One possible solution for such cases could be to transform the molecule of interest into a compound *in vivo* that is better tolerated by the cell. The prerequisites of this strategy are to have a high-capacity, unsaturated pathway for transformation as well as a sensory device for the downstream product.

To characterize further the dynamic response of the sensor, we proposed and validated a second-order model linking biomass to fluorescence. We showed that dynamic parameters were specific to each construct and that they were not significantly altered by changes in the production rate of malonyl-CoA. This result shows the robustness of the sensor for its use in pathway regulation, since a dynamic response uncoupled from the measured signal is often necessary in order to assure the stability of the feedback loop. Moreover, in such regulation strategies, knowledge of the sensor’s parameters is of critical importance in the choice of one particular control architecture over another (Stevens and Carothers, 2014). Therefore, we hope our effort of modeling experimental data provided by this malonyl-CoA sensor will facilitate future developments in dynamic regulation of pathways such as the ones involved in fatty acid or flavonoid production.

Our most unexpected findings arose when we investigated the effect of providing the substrates of malonyl-CoA production on the fluorescence levels produced by the sensor. On one hand, providing Na-malonate to *matB*, resulted in an increased fluorescence, possibly indicating more product formation. On the other hand however, administering β-alanine or Na-malonate to *mmsA* and *atoDA*, respectively resulted in an opposite effect. This phenomenon was especially pronounced for *atoDA*, which was notably the most effective malonyl-CoA producer among the constructs tested in this study. We can only speculate on the mechanism, which may even be a direct effect on the sensor plasmid, and may not represent an actually reduced malonyl-CoA synthesis. According to our hypothesis, it could be due to the fact that *atoDA* (as well as *matB*) are consumers of cellular acetyl-CoA, thereby causing an energy depletion. This is probably more severe if we provide the missing substrate (malonate), with very high enzyme concentrations, and thereby push the reaction toward the product. Perhaps, this energy depletion inhibits RFP production more than the newly produced malonyl-CoA would elevate it. Clearing this issue nevertheless requires further experiments.

When summarizing the observed results, several conclusions important to the metabolic engineer in general can be drawn. First, it is useful to test the performance of several parallel clones after transformation of the constructs, and choose the best for further experiments. Second, the reproducibility of cultures re-grown from glycerol stocks is acceptable, but repeated measurements are advisable to uncover the repeated emergence of deleterious mutants, possibly indicating genetic instability. Third, this instability was minimal when using the lowest copy-number vector for expressing the producer construct. This provides a further argument to start testing various alternative enzymes by expressing them from low-copy vectors with gradually controllable promoters, and varying ribosome binding sites to increase expression and find a construct that is optimal both in efficiency and stability. And finally, the variation among measurements obtained in the same run were small enough to test the effect of adding the substrates of the tested pathways to the cell culture, and obtain the optimal IPTG levels for most effective utilization. This approach could be useful in the future to optimize the substrate concentration itself, as well as any other component of the culture medium or the induction process in a high throughput, combinatorial fashion.

## Conflict of Interest Statement

The authors certify that there is no conflict of interest with any financial organization regarding the material discussed in the manuscript. The Associate Editor, Jean Marie François, declares that, despite hosting a Frontiers Research Topic alongside the author Pablo Carbonell, the review process was handled objectively and no conflict of interest exists.

## Supplementary Material

The Supplementary Material for this article can be found online at http://journal.frontiersin.org/article/10.3389/fbioe.2015.00046
