# Supplementary material for: A Sense of Balance: Experimental Investigation and Modeling of a Malonyl-CoA Sensor in Escherichia coli
Source: Front Bioeng Biotechnol. 2015 Apr 8;3:46. doi: 10.3389/fbioe.2015.00046 (PMC4389729; doi:10.3389/fbioe.2015.00046)
Supplement: Supplementary file 1 [file Data_Sheet_1.PDF]

Supplementary Material

A sense of balance: Experimental investigation and modeling of a malonyl-CoA sensor in *Escherichia coli*

Fehér, Tamás; Libis, Vincent; Carbonell, Pablo; Faulon, Jean-Loup

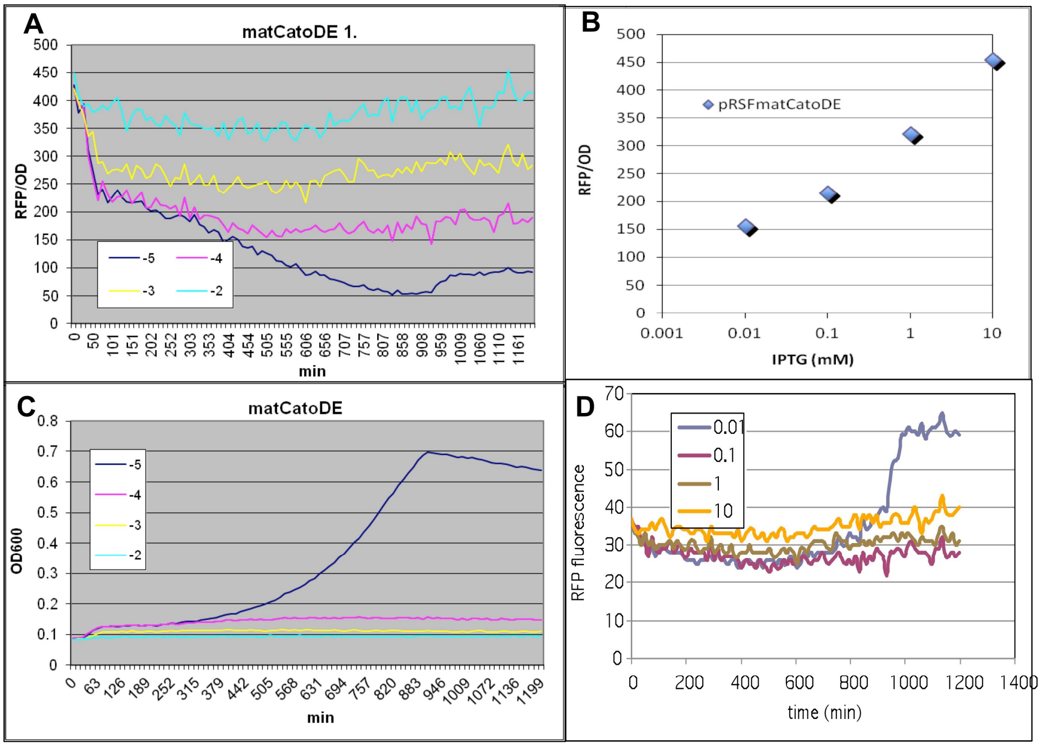

Figure S1. Uncovering an artifact. (A) OD-normalized fluorescence evolution upon induction of pRSF*matCatoDA* with various IPTG concentrations, (B) peak fluorescence vs. IPTG concentration, (C) optical density vs. time and (D) RFP fluorescence vs. time. The seeming IPTG-dependent fluorescence levels produced by this construct, seen on (A) and (B) are falsified by the lack of growth at most IPTG concentrations, and the lack of increase of fluorescence levels on panels (C) and (D), respectively.

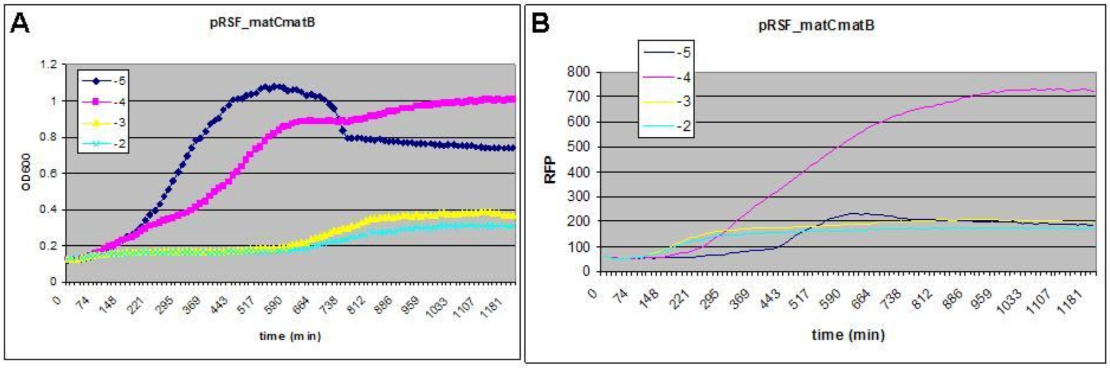

Figure S2. Observing a loss of fluorescent protein production. E. coli cells carrying the pCFR sensor and the pRSFmatCmatB production plasmid were induced with IPTG of 10^{-5}, 10^{-4}, 10^{-3} and 10^{-2} M. (A) OD and (B) RFP fluorescence was plotted versus time. A sudden increase in the growth of the samples induced with 10^{-3} and 10^{-2} M can be seen in between 600 and 800 min, without any change in fluorescence, possibly indicating a mutation leading to the loss of production. Na-malonate was included in all samples.

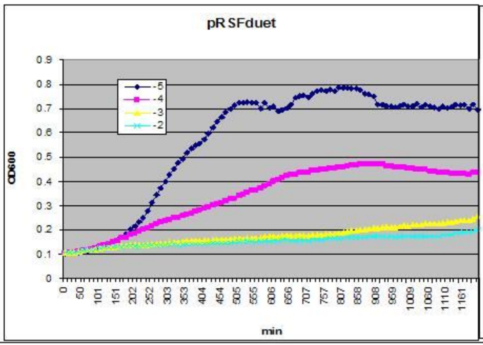
pRSFduet
OD600
min
-5
-4
-3
-2

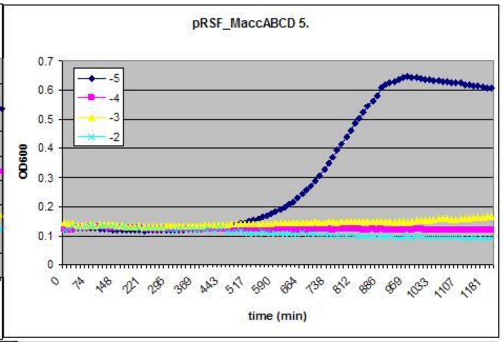
pRSF_MaccABCD 5.
OD600
time (min)
-5
-4
-3
-2

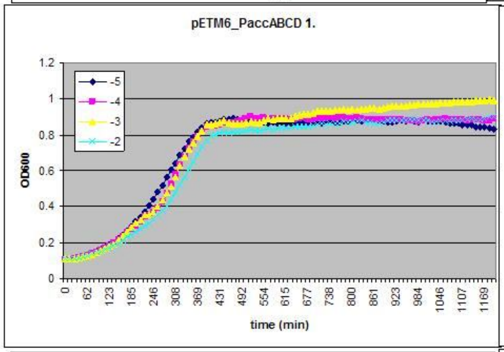
pETM6_PaccABCD 1.
OD600
time (min)
-5
-4
-3
-2

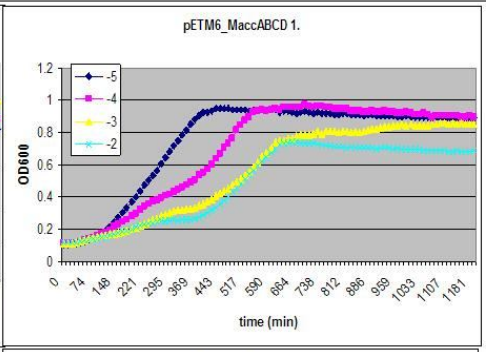
pETM6_MaccABCD 1.
OD600
time (min)
-5
-4
-3
-2

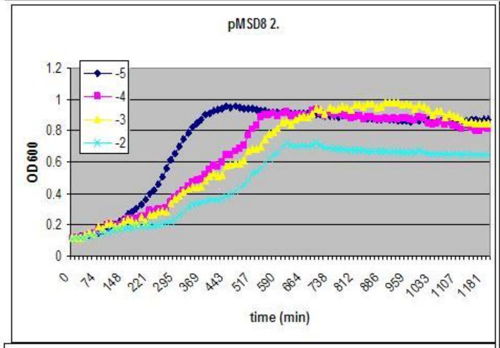
pMSD8 2.
OD600
time (min)
-5
-4
-3
-2

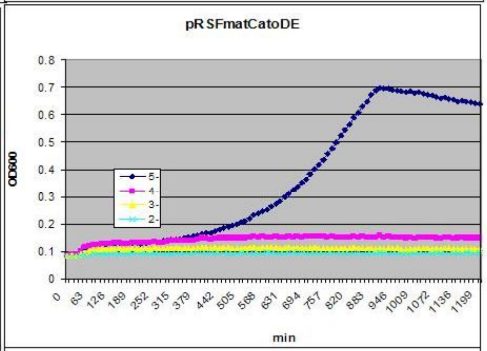
pRSFmatCatoDE
OD600
min
5-
4-
3-
2-

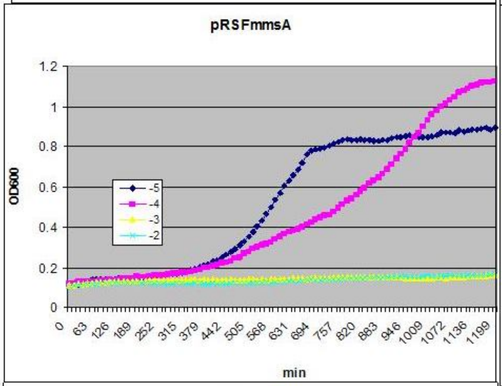
pRSFmmsA
OD600
min
-5
-4
-3
-2

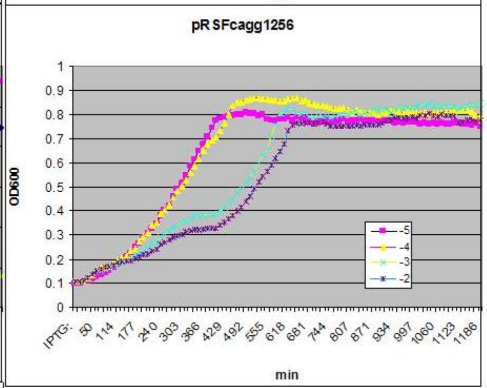
pRSFcagg1256
OD600
min
IPTG:
-5
-4
-3
-2

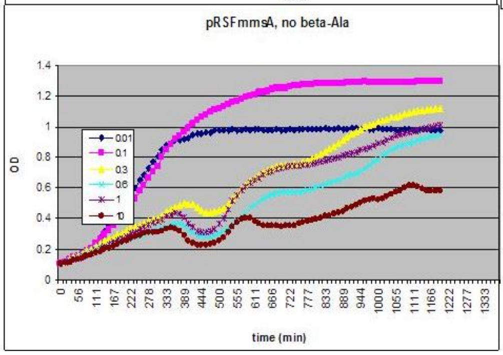
pRSFmmsA, no beta-Ala
OD
time (min)
001
01
03
06
1
10

Figure S3A. Growth curves of *E. coli* BL21DE3 cells carrying the indicated producer plasmid, along with pCFR, induced with 10^{-5}, 10^{-4}, 10^{-3} or 10^{-2} M IPTG.

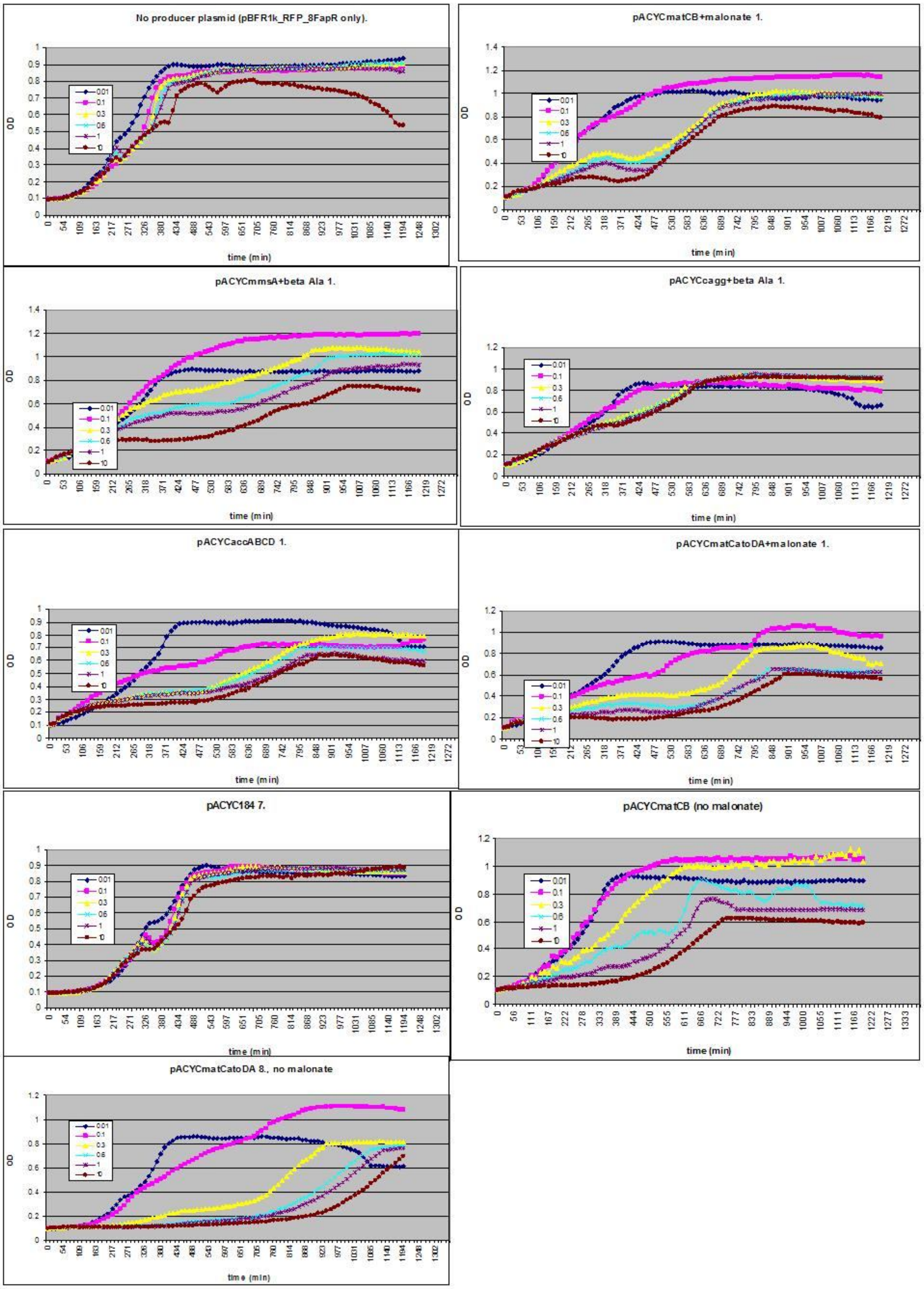

Figure S3B. Growth curves of *E. coli* BL21DE3 cells carrying the indicated producer

plasmid, along with pBFR1k_RFP_8FapR, induced with 0.01, 0.1, 0.3, 0.6, 1, or 10 mM IPTG.

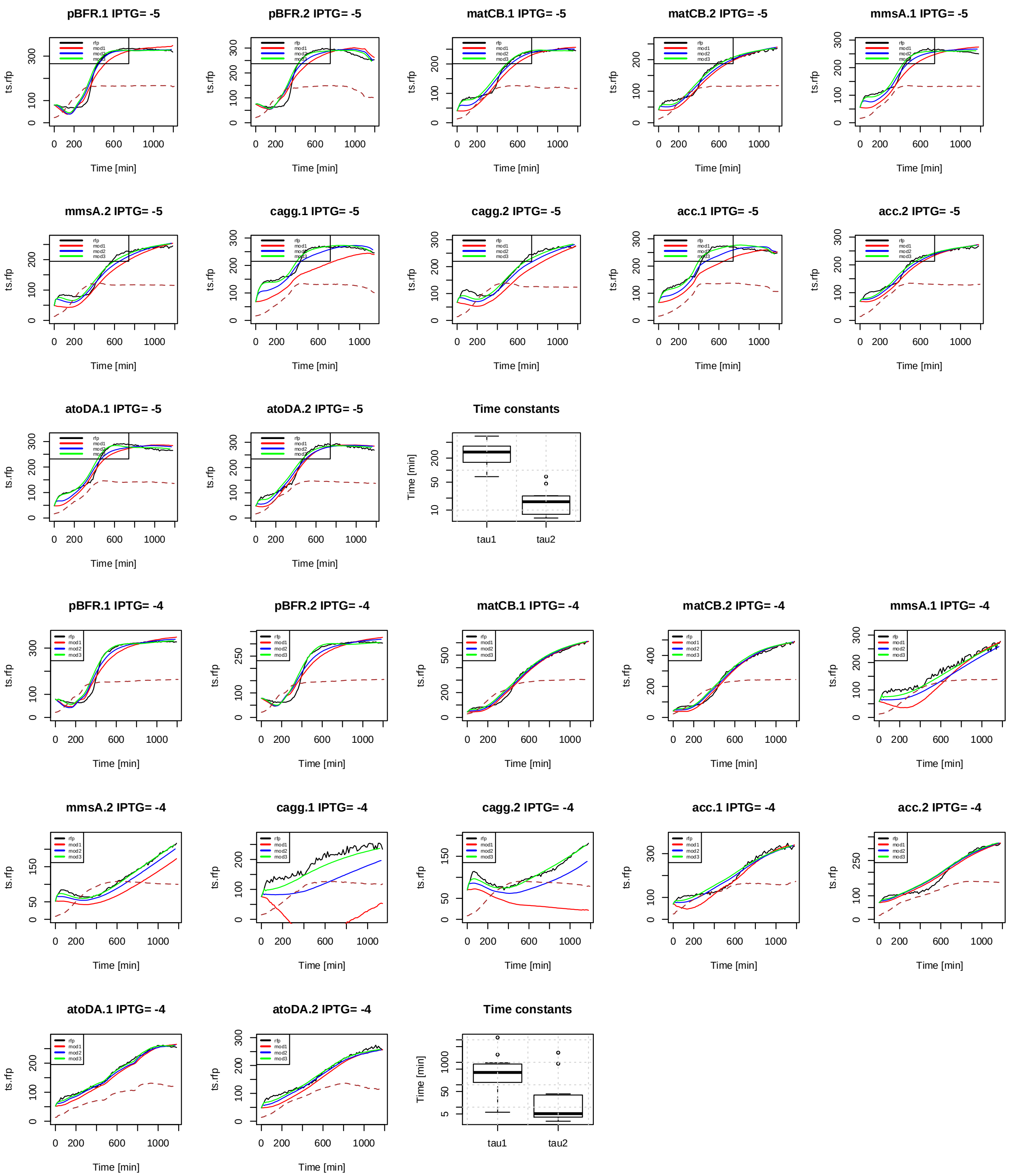

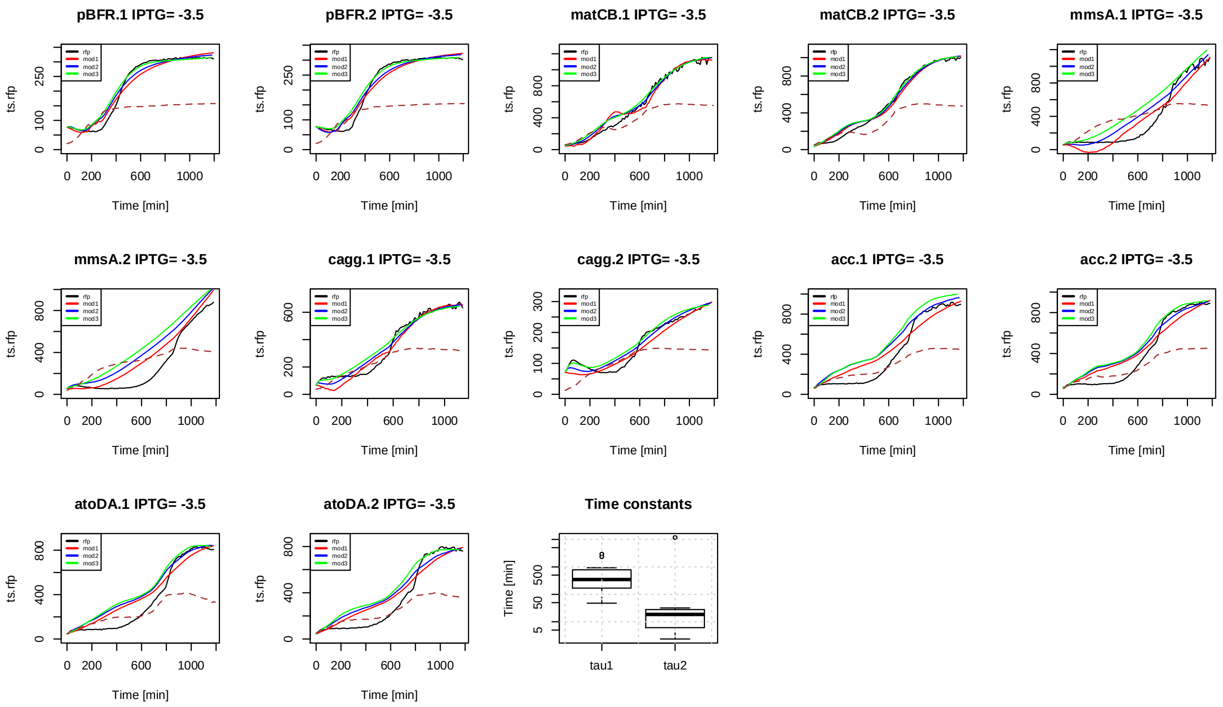

pBFR.1 IPTG= -3.5
pBFR.2 IPTG= -3.5
matCB.1 IPTG= -3.5
matCB.2 IPTG= -3.5
mmsA.1 IPTG= -3.5
mmsA.2 IPTG= -3.5
cagg.1 IPTG= -3.5
cagg.2 IPTG= -3.5
acc.1 IPTG= -3.5
acc.2 IPTG= -3.5
atoDA.1 IPTG= -3.5
atoDA.2 IPTG= -3.5
Time constants
ts.rfp
Time [min]
rfp
mod1
mod2
mod3
tau1
tau2

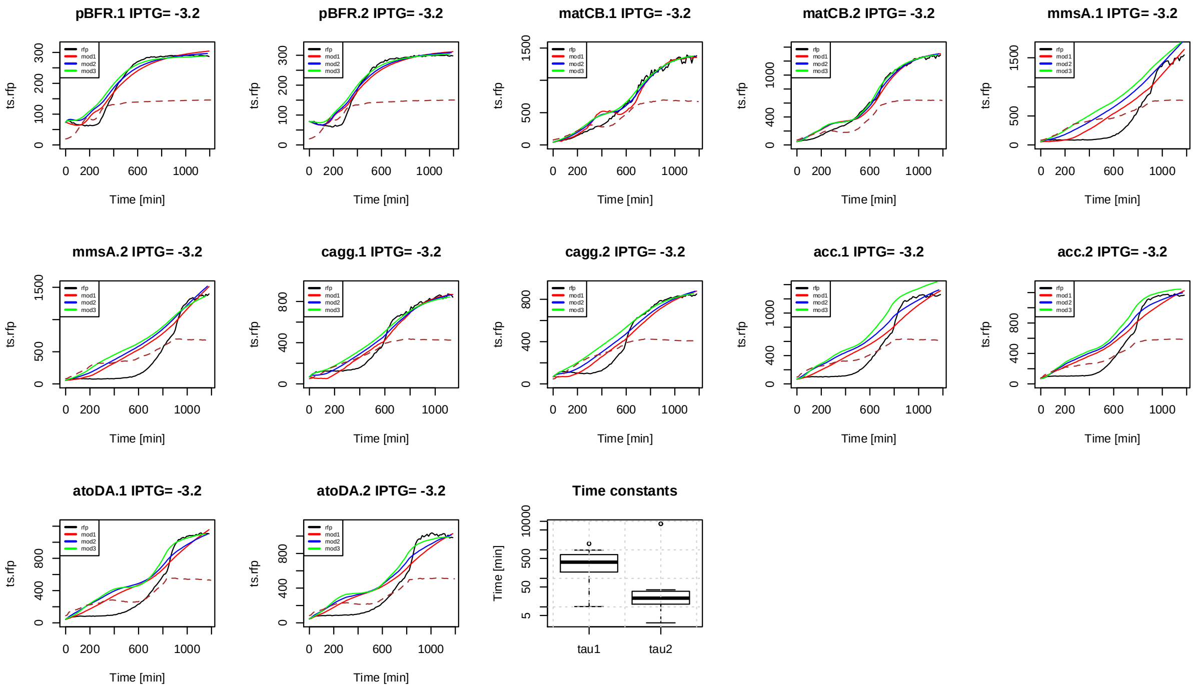

pBFR.1 IPTG= -3.2
pBFR.2 IPTG= -3.2
matCB.1 IPTG= -3.2
matCB.2 IPTG= -3.2
mmsA.1 IPTG= -3.2
mmsA.2 IPTG= -3.2
cagg.1 IPTG= -3.2
cagg.2 IPTG= -3.2
acc.1 IPTG= -3.2
acc.2 IPTG= -3.2
atoDA.1 IPTG= -3.2
atoDA.2 IPTG= -3.2
Time constants
ts.rfp
Time [min]
rfp
mod1
mod2
mod3
tau1
tau2

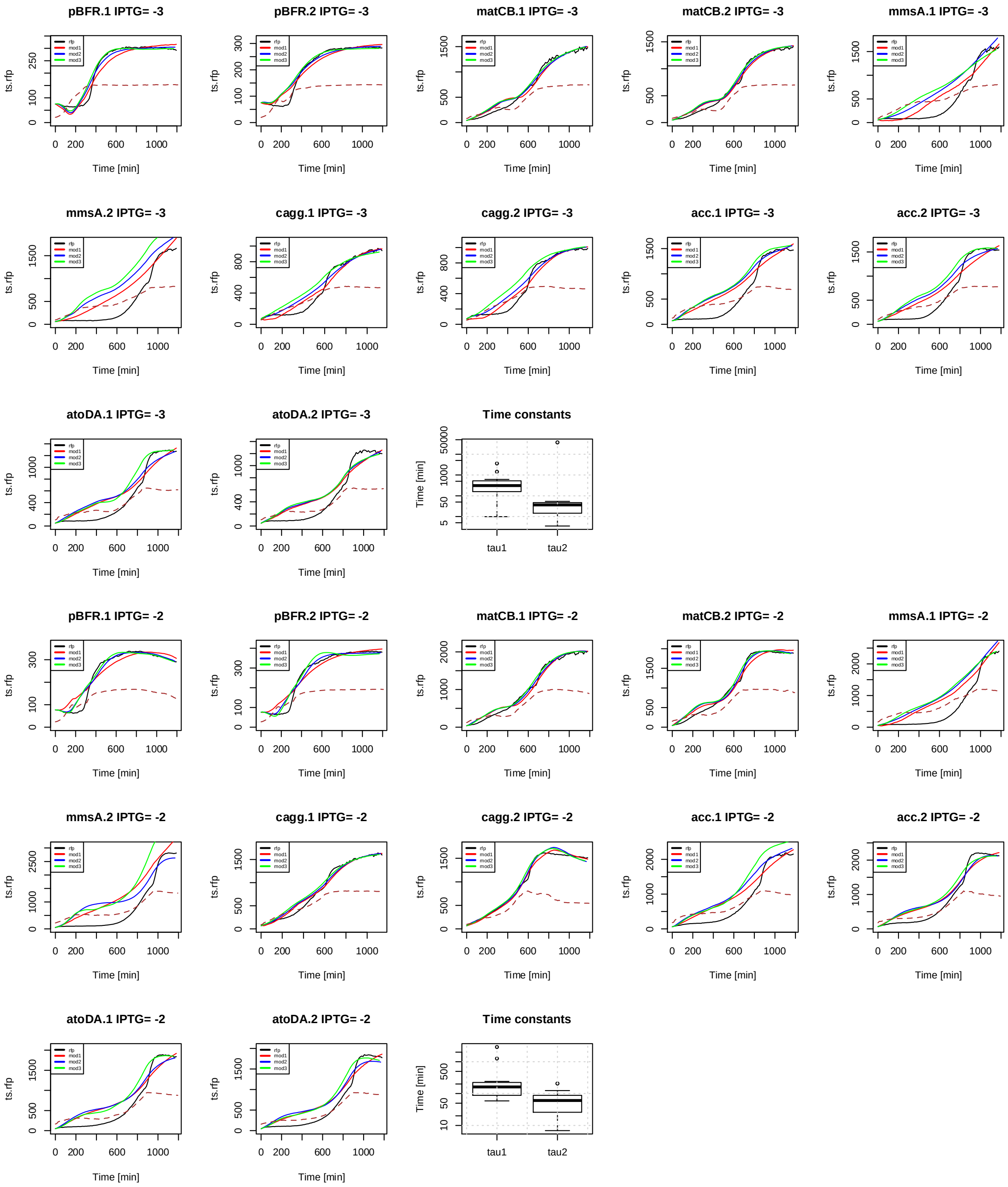

Figure S4. Fitting of the measured RFP fluorescence response to the three models. BL21DE3 cells carrying the pBFR1k_RFP_FapR sensor plasmid alone (marked pBFR), or accompanied by the producer plasmid pACYC*matCmatB* (marked matCB), pACYC*mmsA* (represented as mmsA) pACYC*cagg1256* (depicted as cagg), pACYCM*accABCD* (acc) or pACYC*matCatoDA* (atoDA) were each induced with IPTG concentrations of 0.01 mM, 0.1 mM, 0.3 mM, 0.6 mM, 1 mM, and 10 mM, and the evolution of absolute fluorescence was plotted against time. Na-malonate was added for pACYC*matCmatB* and pACYC*matCatoDA*, and β-alanine was administered for pACYC*mmsA* and pACYC*cagg1256*. Two replicas were carried out for each measurement, indicated by the number after the construct name. IPTG

concentration is shown as a $\log_{10}$ value, in mol/L. The distributions of the fitted two time constants for all constructs are shown in a boxplot.

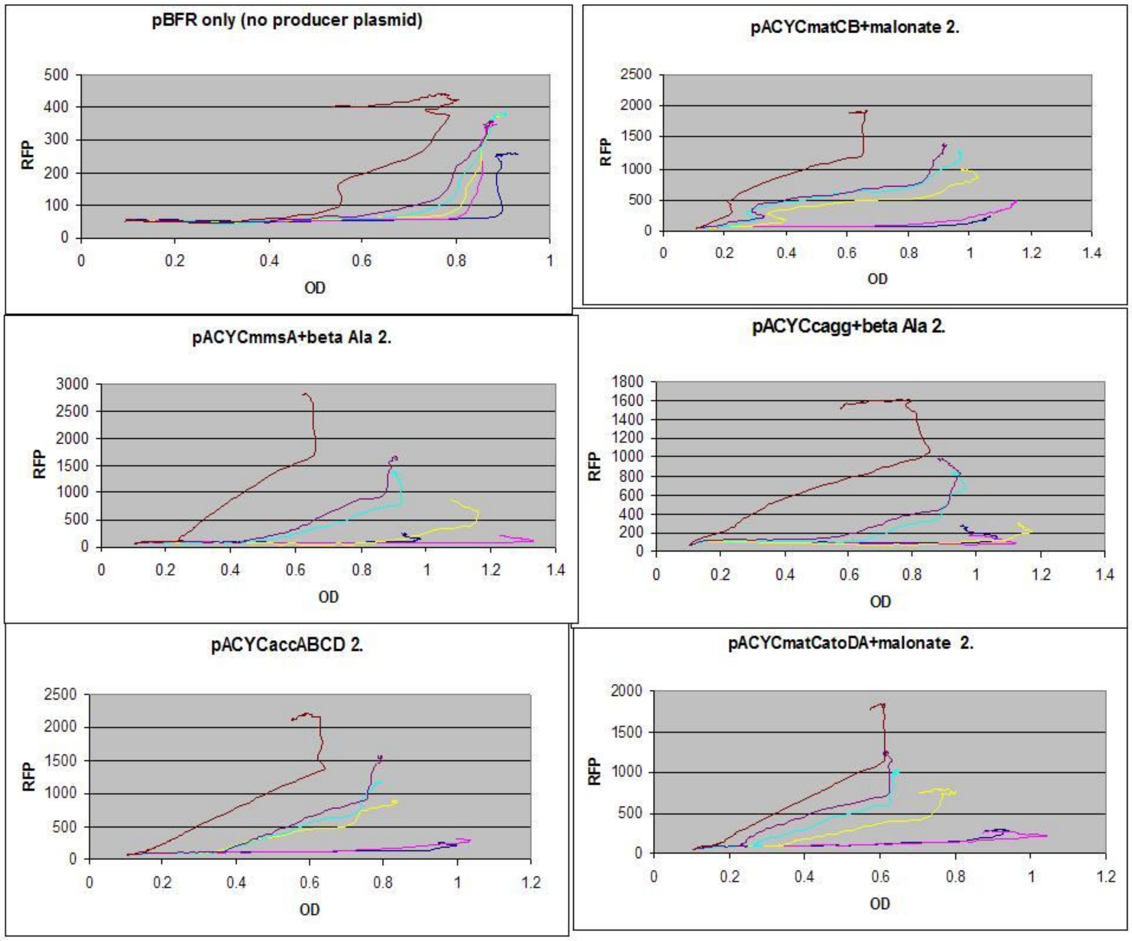

Figure S5. RFP fluorescence vs. OD plots of various *E. coli* BL21DE3 cells carrying the indicated producer plasmid. Colors indicate the concentration of IPTG used for induction: dark blue: 0.01 mM; magenta: 0.1 mM; yellow: 0.3 mM; cyan: 0.6 mM; purple: 1 mM; brown: 10 mM

**plasmid name**	**rep. origin**	**resistance**	**function**	**reference**
pBFR1k_RFP_8FapR	BBR1	Km	sensor	Liu et al, 2013
pCFR	BBR1	Cm	sensor	this work
pRSF*mmsA*	RSF	Km	producer	Feher et al., 2014
pRSF*cagg1256*	RSF	Km	producer	Feher et al., 2014
pRSF*matCmatB*	RSF	Km	producer	Wu et al., 2013
pRSFM*accABCD*	RSF	Km	producer	Feher et al., 2014
pRSF*matCatoDA*	RSF	Km	producer	Feher et al., 2014
pMSD8	pSC101	Ap	producer	Davis et al., 2000
pETM6-M*accABCD*	pColE	Ap	producer	Xu et al., 2013
pETM6-P*accABCD*	pColE	Ap	producer	Xu et al., 2013
pACYC*mmsA*	p15A	Cm	producer	this work

pACYC*cagg1256*	p15A	Cm	producer	this work
pACYC*matCmatB*	p15A	Cm	producer	Wu et al., 2013
pACYCM*accABCD*	p15A	Cm	producer	this work
pACYC*matCatoDA*	p15A	Cm	producer	this work

Table S1. Plasmids used in this study.

**Sensed with pBFR1k_RFP_8FapR**	**CV (%)**	**Sensed with pCFR**	**CV (%)**
pACYC*cagg1256*	34	pRSF*cagg1256*	43
pACYC*matCmatB*	18	pRSF*matCmatB*	41
pACYC*mmsA*	50	pRSF*mmsA*	75
pACYCM*accABCD*	19	pRSFM*accABCD*	ND
pACYC*matCatoDA*	15	pRSF*matCatoDA*	ND
pMSD8	9	pMSD8	13
pETM6-M*accABCD*	72	pETM6-M*accABCD*	20
pETM6-P*accABCD*	53	pETM6-P*accABCD*	47

Table S2. The coefficient of variation (CV) of fluorescence values obtained at OD=0.6 for cells carrying the indicated producer plasmid when induced with 1 mM IPTG. The sensor plasmid used for the measurement is shown on top.
